# Socio-ecological determinants of multiple anthropometric failures among under-five children: A systematic review and meta-analysis of observational studies

**DOI:** 10.1371/journal.pgph.0005008

**Published:** 2025-07-31

**Authors:** Biniyam Sahiledengle, Paul Russell Ward, Bereket Duko, Kingsley Emwinyore Agho, Lillian Mwanri

**Affiliations:** 1 Department of Public Health, Madda Walabu University Goba Referral Hospital, Bale-Goba, Ethiopia; 2 Research Centre for Public Health Research, Equity and Human Flourishing, Torrens University Australia, Adelaide, South Australia, Australia; 3 School of Health Sciences, Western Sydney University, Penrith, New South Wales, Australia; PLOS: Public Library of Science, UNITED STATES OF AMERICA

## Abstract

The composite index of anthropometric failure (CIAF) offers a comprehensive measure of the overall burden of undernutrition in children, extending beyond the traditional anthropometric indices to better capture the co-occurrence of multiple anthropometric deficits. Despite its growing use, evidence on the determinants of CIAF remains fragmented and inconclusive. This systematic review and meta-analysis aimed to identify and synthesize the determinants of CIAF among under five children. A comprehensive search of nine major databases was conducted, including MEDLINE (PubMed), Embase (Ovid), Scopus, CINAHL, ProQuest, ScienceDirect, Global Index Medicus, the Cochrane Library, and Google Scholar. Determinants were categorized using a socio-ecological model across intrapersonal, interpersonal, and community levels. Random-effects meta-analyses were conducted to generate pooled odds ratios (ORs), and heterogeneity was assessed using the I² statistic and Cochran’s Q test. Subgroup analyses, sensitivity testing, and publication bias assessment were also performed. Of 6,816 records identified, 56 studies met inclusion criteria (encompassing a total of 1,029,452 under five children). Intrapersonal factors significantly associated with higher odds of CIAF included male sex (OR: 1.17, 95% CI:1.04-1.30), older child age (OR: 1.50, 95% CI: 1.42-1.59), diarrhea (OR: 1.18, 95% CI: 1.08-1.29), fever (OR: 1.08, 95% CI: 1.04-1.13), anemia (OR: 1.22, 95% CI: 1.16-1.29), low birthweight (OR: 2.07, 95% CI: 1.51-2.83), and poor dietary diversity (OR: 1.11, 95% CI: 1.06-1.17). Interpersonal and community-level determinants significantly associated with increased odds of CIAF included low maternal education, maternal unemployment, household poverty, larger family size, food insecurity, and use of unimproved drinking water. We identified key modifiable risk factors associated with CIAF among under five children at different levels, including inadequate dietary intake, childhood morbidity, household food insecurity, limited maternal education, and poor access to safe water. These findings emphasize the need for comprehensive, multi-level interventions that address modifiable risk factors across individual, household, and community levels to reduce childhood multiple anthropometric failures.

## Introduction

Childhood undernutrition, which includes underweight, wasting, and stunting is a key indicator of child growth and development [1]. Wasting (low weight for height) reflects acute malnutrition, underweight (low weight for age) reflects both acute and chronic malnutrition and stunting (short height for age) indicates of chronic malnutrition [1,2]. Children suffering from undernutrition often encounter both immediate and long-term negative effects on their physical health and cognitive development [3]. Over the last decade, there have been promising results in reducing childhood undernutrition on a global scale (4). However, undernutrition remains a pressing public health challenge in many Low- and Middle-Income Countries (LMICs), despite its overall decline [4,5]. Moreover, children in LMICs increasingly experience multiple anthropometric failures, with alarmingly high prevalence rates [6,7]. This increase significantly hinders the United Nations’ global commitment to ending all forms of undernutrition (Sustainable Development Goal (SDG)- 2, Target 2.2) by 2030 [8].

Traditional anthropometric indices (i.e., underweight, wasting, and stunting) are widely used to assess childhood nutritional status; however, they do not capture the full extent of multiple anthropometric failures [9]. In many instances, different forms of undernutrition occur simultaneously in a single child [10–13]. A child can exhibit one or more of these conditions concurrently, these indices fail to provide a comprehensive view of the overall nutritional burden in children. To address this, the Composite Index of Anthropometric Failure (CIAF) combines these three forms of undernutrition into a single index, providing a more comprehensive assessment of overall undernutrition in children [9,14]. The CIAF categorizes children into groups based on their nutritional status (stunting only, wasting only, underweight only, a combination of stunting and underweight, a combination of wasting and underweight, and a combination of all three conditions (stunting, wasting, and underweight) simultaneously).This approach provides a more accurate estimate and identifies undernourished children who individual indicators might overlook [9,14].

Multiple anthropometric failures in children result from a complex interplay of nutritional, demographic, and socio-economic factors [13,15–18]. Major contributors include poverty, food insecurity, inadequate dietary intake, illness, limited healthcare access, and poor water, sanitation, and hygiene (WASH) conditions [19–29]. These factors create a vicious cycle, complicating efforts to address childhood undernutrition. While global initiatives such as the SDGs guide nutritional policies, a gap exists in understanding the drivers of multiple anthropometric failures in children. A comprehensive understanding of these determinants is crucial for addressing undernutrition and improving children’s long-term health [30].

There is a growing body of literature on childhood undernutrition using the CIAF across diverse settings [19,20,31,32]. Existing studies have identified several potential drivers of CIAF, including maternal characteristics, socio-economic status, nutritional factors, child-related attributes, and environmental conditions [19,22,23,25–27,31,33,34]. However, most available studies are localized, conducted at regional or local levels within specific countries, which limits their generalizability. Moreover, findings across these studies have been inconsistent, and there is a lack of comprehensive synthesis to integrate the evidence [19–21,31–33,35–37]. This highlights the need for a comprehensive systematic review to synthesize the existing evidence on the determinants of CIAF.

To date, no systematic review or meta-analysis has yet synthesized evidence on the determinants of undernutrition as measured by the CIAF in children under five [38–42]. Unlike single-metric approaches that assess only chronic (stunting) or acute (wasting) undernutrition, CIAF captures all anthropometric failures stunting, wasting, underweight, and their combinations revealing a wider range of determinants, often overlooked in isolated analyses. By aggregating overlapping conditions, CIAF avoids double-counting, identifies coexisting vulnerabilities, and provides a more comprehensive estimate [9,14]. To our knowledge, this is the first systematic review and meta-analysis to comprehensively identify and summarize evidence on the potential determinants of CIAF among children under five. Identifying these determinants will support global efforts to achieve the SDGs-particularly the goal of ending childhood malnutrition and will aid in developing more targeted interventions to reduce the burden of CIAF.

## Methods

### Data sources and searching strategies

This systematic review and meta-analysis followed the Preferred Reporting Items for Systematic Reviews and Meta-Analysis (PRISMA) guideline (S1 Checklist) [43]. We performed comprehensive search across nine major databases and search engines: Embase/Ovid, MEDLINE (PubMed), Scopus, Cumulative Index to Nursing, and Allied Health Literature (CINAHL)/EBSCO, ProQuest (EBSCO), Science Direct, Global Index Medicus, and the Cochrane Library. A search was also undertaken in Epistemonikos a systematic review’s repository. To incorporate non-indexed studies, we conducted searches using internet search engines, including Google and Google Scholar, to ensure a comprehensive review of relevant literature. We also screened the reference lists of included studies for additional articles. To ensure comprehensive coverage of relevant literature following the introduction of the Composite Index of Anthropometric Failure (CIAF) by Svedberg in 2000 [9], studies published between January 1, 2000, and December 31, 2024, were included. No language restrictions were applied during the selection of studies published within this period. The initial search was conducted on November 27, 2024, with weekly updates performed through December 31, 2024. The protocol for the study was prospectively registered in the International Prospective Register of Systematic Reviews (PROSPERO: CRD42022354416), ensuring compliance with systematic review standards. The search strategy utilized a combination of carefully selected keywords and Medical Subject Headings (MeSH) terms, including but not limited to the following: “child” [MeSH Terms], “child, preschool” [MeSH Terms], “under-five child*”, “risk factors” [MeSH Terms], “determinant*”, “determinant factors”, “associated factors”, “factor*”, “prevalence and associated factors”, “association”, “risk”, “predictor*”, “child nutrition disorders” [MeSH Terms], “malnutrition” [MeSH Terms], “undernutrition”, “stunting*”, “wasting”, “underweight”, “nutritional status” [MeSH Terms], “nutrition assessment” [MeSH Terms], “anthropometric failure”, “anthropometric index”, “aggregate*”, “coexist*”, “concurrent*”, and “CIAF”, using appropriate Boolean operators (AND, OR) and truncation. The full lists of keywords and medical subject headings (MeSH) terms and along with specific database search terms used in this review are available in the S1 Text.

### Eligibility criteria

The Population, Concept, and Context (PCC) framework was employed as the most suitable approach for this systematic review, as it enables a comprehensive exploration of the broader determinants of CIAF [44]. With regards to PCC framework, P stands of populations under review (under-five children), C stands for concept (determinants of CIAF) and the second C stands for context). No geographic and language restrictions were applied, aiming to capture global evidence from peer-reviewed journal articles. All observational studies (i.e., cross-sectional, case-control, and cohort studies), as well as randomized and/or quasi-experimental studies reporting the determinants, predictors, or risk factors for CIAF among children under five, were considered for inclusion. Studies that reported effect size or estimate, such as adjusted odds ratio (AOR) or prevalence ratio (PR) or risk ratio (RR) were included in the meta-analysis. However, qualitative studies, policy papers, opinion pieces, case studies, case series, case reports, letters to the editor, short communications, reviews, commentaries, protocols, conference proceedings, scoping reviews, rapid reviews, and systematic reviews and meta-analyses were excluded. Studies focusing solely on undernourished children without control groups (e.g., well-nourished children or those without anthropometric failure) were also excluded. Among the studies that analyzed the factors associated with CIAF using similar datasets such as the Demographic and Health Surveys (DHS), the study that offered the most comprehensive analysis was selected for inclusion in this systematic review.

### Study selection and data extraction

First, all articles were imported to EndNote 20 to remove duplicates. Then, the remaining records were exported to Covidence for title, abstract, and full text screening. Two authors (BS and LM) independently assessed eligibility and conducted the full-text review using Covidence, based on predefined inclusion and exclusion criteria. Any discrepancies were resolved through discussion between the two authors, with a third investigator (PW) involved, if necessary, to resolve disagreements. A spreadsheet was created to extract relevant data from each included study, capturing key factors associated with the outcomes of interest. The extracted data included the following key variables: name of the first author, year of publication, country, study design, study setting, geographic focus (local area, national, or multi-country), sample size prevalence of CIAF, determinants of CIAF included in the fully adjusted model, adjusted odds ratios (AOR) or relative risk (RR) or prevalence ratio (PR) with their corresponding 95% confidence interval (CI), and the confounders controlled for in each study.

### Risk of bias (quality) assessment

The modified version of the Newcastle-Ottawa Scale (NOS) for cross-sectional studies was used to assess study quality [45]. The NOS evaluates studies based on three categories: selection, comparability, and outcome. The selection category assesses the representativeness of the sample, sample size, non-respondent, and ascertainment of the exposure (risk factors). The comparability category evaluates whether confounding factors are controlled, while the outcome category focuses on the assessment of outcomes and the appropriateness of statistical tests used. Studies were scored based on these criteria, with a maximum of 10 points. Studies scoring 9–10 points were classified as “Very Good,” 7–8 points as “Good,” 5–6 points as “Satisfactory,” and 0–4 points as “Unsatisfactory.”

### Strategy for data synthesis

A descriptive summary was used to present the characteristics of the included studies. To examine the key determinants of CIAF, confounder-adjusted odds ratios (OR), relative risk (RR), and prevalence ratios (PR) were used. The OR estimate from primary studies were pooled using the generic inverse variance method, which involved converting the AOR to a logarithmic scale and then calculating standard error (SE) based on the 95% confidence intervals. Since the reference categories in few studies differ, we computed the reciprocal of the adjusted odds ratios for studies where the reference category needed to be reversed to ensure consistency and uniformity for binary categorical predictors [46]. Inverse variance weighted random-effect meta-analyses, using the DerSimonian and Laird method, were applied to estimate the pooled odds ratio (OR) with its 95% CI for determinants associated with CIAF, which accounts for the heterogeneity between studies [47]. To assess magnitude of statistical heterogeneity across included studies the Cochran’s Q- and I²- statistics were used. According to Higgins et al. (2003), heterogeneity was categorized based on I² as low (0–24%), moderate (25–49%), high (50–74%), and very high (≥ 75%) [48]. The CIAF determinants were classified into three levels based on the adapted socio-ecological framework: intrapersonal (child-related), interpersonal (parental and household-related), and community [49,50]. Subgroup analyses were conducted as sensitivity tests to assess the robustness of the estimates and explore the potential source of heterogeneity for each factor. Potential publication bias was assessed using Egger’s regression test for asymmetry and by inspection of the funnel plot. A leave-one-out sensitivity analysis was conducted to determine the influence of individual studies on pooled estimates. All statistical analyses were performed using STATA 17 (StataCorp LLC, Stata Statistical Software: Release 17; College Station, TX, USA, 2021).

### Operational definition

Composite index of anthropometric failure (CIAF) categorizes children into seven mutually exclusive groups based on their anthropometric status: (A) no failure; (B) wasting only; (C) wasting and underweight; (D) wasting, stunting, and underweight; (E) stunting and underweight; (F) stunting only; and (Y) underweight only. A child is considered undernourished according to the CIAF if they fall into any of the failure categories (B–Y) (14). Anthropometric failures were defined using the World Health Organization (WHO) Child Growth Standards as follows: Stunting is defined as a height-for-age Z-score (HAZ) of less than -2 standard deviations (SD) from the WHO growth standard. Wasting is classified as a weight-for-height Z-score (WHZ) below -2 SD from the WHO growth standard. Underweight is determined by a weight-for-age Z-score (WAZ) of less than -2 SD from the WHO growth standard [51].

## Results

### Identification of studies

**Fig 1:**
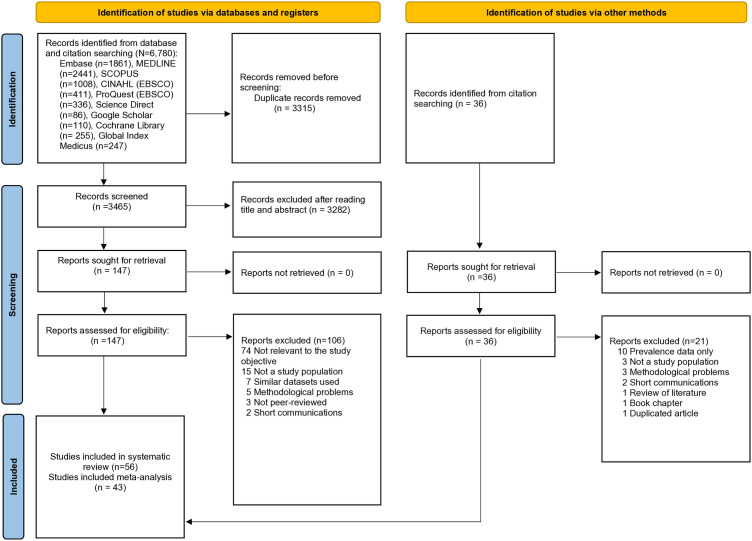
A PRISMA flow diagram illustrating the process of systematic review and meta-analysis.

Our initial search yielded 6,816 records, including 36 identified through citation searching, from which 3,315 duplicates were removed. Title and abstract screening excluded 3282 studies, leaving 183 articles for full-text assessment and eligibility. After full-text review, a total of 56 articles were included in this systematic review and meta-analysis. Of these, 43 studies were included in the meat-analysis (Fig 1). S1 Table provides detailed characteristics of the articles excluded after full-text review, along with the specific reasons for their exclusion from the systematic review and meta-analysis.

### Description of included studies

The general characteristics of the included studies are summarized in Table 1. Overall, the systematic review incorporated 56 studies (55 cross-sectional and one cohort study) from twelve different countries, encompassing a total of 1,029,452 under-five children. Of these, 35 studies were conducted in Asia (Bangladesh, India, and Pakistan), 14 in Africa (Ethiopia, Nigeria, and Tanzania), and 4 in the East Asia and Pacific regions (China, Indonesia, Malaysia, and Thailand), two in South America (Argentina), and one study from Middle East (Yemen). The majority of primary studies originated from India (n = 21) [22,34,52,53], followed by Ethiopia (n = 11) [24,27,29,54–61], Pakistan (n = 7) [23,62–67], Indonesia (n = 4) [21,68–70], Bangladesh (n = 3) [26,71,72], Nigeria (n = 2) [73,74], and Argentina (n = 2) [75,76]. One study each was included from Tanzania [77], China [78], Malaysia [79], Thailand [80], and Yemen [81]. Additionally, one multi-country study conducted in Bangladesh and India was also included (n = 1) [82]. Sample sizes varied substantially, ranging from 141 [70] to 210,977 participants [22]. In terms of CIAF prevalence, the highest rate was reported in Bangladesh (Asia) 78.4% [26], while the lowest was observed in South America (Argentina) 21.3% [76].

**Table 1:** Descriptive summary of included studies in the systematic review and meta-analysis, 2000-2024.

Authors name, Year of publication	Study design	Age in months	Sample size	Country	Region	Geographic focus	Prevalence of CIAF	Adjusted confounders	Protective/risk factors identified
Shah G et al, 2024 [22]	CS	0 to 59 months	210,977	India	Asia	National	52.6	Birth order, consumption of fresh milk, consumption of formula milk, postnatal baby checkup, mother’s age, employment status, mode of delivery (c-section), mother’s anemia level, type of cooking fuel used, water source, father’s education level, religion	Sex, age, childbirth size, initiation of breastfeeding, breastfeeding, mother’s BMI, mother education, wealth index, place of residence, toilet facility, milk consumption, breastfeeding duration
Kundu RN et al, 2023 [52]	CS	0 to 59 months	197090	India	Asia	National	52.2	Sex of the child, parents alive, birth order, social category, wealth index	Mother’s education, family size, child’s anemia level
Soni A et al, 2022 [121]	CS	0 to 36 months	187452	India	Asia	National	61.4	Number of siblings, ethnic group, access to toilet facility, having separate kitchen, type of cooking fuel used, uses of soap after toilet use	Maternal education, sex of the child, birth interval, birthweight
Khamis AG et al, 2020 [77]	CS	0 to 59 months	37,205	Tanzania	Africa	National	45.1	Mothers age at first birth, number of births in the five years, gender of the head of the household, mother’s employment, place of delivery, presence of diarrhea, early initiation of breastfeeding, marital status, age of the head of the household	Residency, mother’s BMI, mother education, wealth status, age of the child, gender, birthweight, symptoms of fever, dietary diversity score
Ghosh P, 2023 [122]	CS	0 to 59 months	31,599	India	Asia	National	60.3	Sex of the child, age of the child, birth size, birth order, breastfeeding, mother’s age, birth in last 3 years, mother’s BMI, mother’s anemia level, antenatal care visits (ANC) during pregnancy, mother’s education level, mother’s media exposure, socio-economic status, place of residence, geographic regions	Diarrhea, fever, child’s anemia level
Porwal A et al, 2021 [113]	CS	0 to 59 months	30,500	India	Asia	National	48.2	Mother’s age, mother’s access to information, parity	Sex of the child, morbidity in past 2 weeks, age, mother’s education, mother’s BMI, place of residence, wealth index, mother’s employment status;
Chowdhury MRK et al, 2021 [71]	CS	0 to 59 months	19,874	Bangladesh	Asia	National	51.9	Underweight mother, birth order, father’s occupation, watching television, wealth index, birth cohort	Age of the child, mother’s working status, mother’s education
Alarape K et al, 2022 [73]	CS	0 to 59 months	19471	Nigeria	Africa	National	40.7	Sex, zone	Age of the child
Naveed TA et al, 2022 [62]	CS	0 to 59 months	14,540	Pakistan	Asia	National	40.5	Wealth index, type of water source, access to toilet facilities, mother education, bank account	Family size
Kundu RN et al, 2022 [82]	CS	0 to 59 months	14055	Bangladesh and India	Asia	Regional	43.3	Religion, household size, age of mother, mode of delivery, antenatal care visits, number of living children, birth order number, place of delivery	Sex of children, age of children, education of mother, mother’s BMI, wealth index, place of residence
Pei L et al, 2014 [78]	CS	0 to 36 months	13,532	China	East Asia & Pacific	Local	21.7	Sex, wealth index, mothers’ age (years), mother education, province, ethnicity	Age of the child, currently breastfeeding
AmusaI LB et al, 2023 [74]	CS	0 to 59 months	10,962	Nigeria	Africa	National	41.3	Sex, size, birth order, vitamin A received, fever, cough, wealth index, mother’s BMI, residence, education, toilet, media exposure	History of diarrhea, fever, maternal employment, currently breastfed, sources of water supply
Sahiledengle B et al, 2024 [29]	CS	0 to 59 months	5259	Ethiopia	Africa	National	42.2	Number of under-five children, vitamin A in last 6 months, minimum meal frequency, age of the mother, ANC visit, place of delivery, source of drinking water, time to water source, region	Sex of the child, age of the child, birth interval, minimum dietary diversity, maternal educational level, wealth index, residence, toilet facility, currently breastfeeding
Ali Khan RE et al, 2014 [72]	CS	0 to 59 months	5258	Bangladesh	Asia	National	54.9	Birth-order of child, age of child, birth-interval, mother’s education, wealth index, breastfeeding, mother’s BMI, cough, number of children under-5 years	Diarrhea, fever, sex of the child, household size
Siddiqa M et al, 2024 [23]	CS	0 to 59 months	4224	Pakistan	Asia	National	45.4	Paternal education, birth order, fresh milk consumed, formula milk consumed, breastfeeding, infant postnatal checkup, maternal age, maternal working status, maternal smoking status, region, iron tablet usage during pregnancy	Sex, age, size of a child at birth, initiation of breastfeeding, mother’s BMI, maternal education, wealth index, residence, toilet facilities, sources of water supply
Endris N et al, 2017 [54]	CS	0 to 59 months	3095	Ethiopia	Africa	National	48.5	Region of residence	Long interpregnancy interval, age of the child, birth interval, maternal education, wealth index
Balogun OS et al, 2021 [63]	CS	0 to 59 months	3071	Pakistan	Asia	National	52.2	Regions, father education, child vaccination, mother’s age at first birth	Residence type, sex of the child, type of toilet facility, socioeconomic status, mother’s education, maternal employment
Das R et al, 2022 [26]	CS	2 to 23 months	1431	Bangladesh	Asia	Local	78.4	Maternal age, the pathogen identified in stool (campylobacter), child age, number of under-5 children at home, toilet facility, type of floor.	Sex of the child, breastfeeding status, maternal education, mother’s BMI, sources of drinking water
Latifah HI et al, 2024 [68]	CS	0 to 23 months	1,327	Indonesia	East Asia & Pacific	Local	24.9	Immunization status, vitamin A administration, deworming, gestational age, parity, sanitation facility, history of pneumonia	Food security, sex of the child, child age, birthweight, history of diarrhea, history of ARI, place of residence, mother’s educational level, economic status
Shahid M et al, 2022 (b) [64]	CS	0 to 36 months	1010	Pakistan	Asia	National	49.4	Sex of the child, size of a child at birth, initiation of breastfeeding, mother’s BMI, maternal education, wealth index, residence, toilet facilities, paternal education, birth order, fresh milk consumed, formula milk consumed, breastfeeding, infant postnatal checkup, maternal age, maternal working status, maternal smoking status, region, iron tablet usage during pregnancy	Age of the child, source of drinking water
Sapkota S et al, 2018 [80]	CS	24 to 59 months	871	Thailand	East Asia & Pacific	Local	27.4	Child age, religion	Maternal employment status
Rathi SK et al, 2024 [123]	CS	0 to 59 months	749	India	Asia	Local	54.5	Age of the child, sex of the child, mothers’ education, monthly income, mother’s BMI, age at which supplementary feedings begin	Mother occupation
Jeyakumar A et al, 2021 [124]	CS	0 to 59 months	577	India	Asia	Local	58.8	Place of delivery, child’s age-appropriate immunization status, colostrum given, timely initiation of complementary feeding after 6 months.	Sex of the child, mother’s education, child age, child’s weight at birth, child diarrhea in last 2 weeks, early initiation of breastfeeding, mother’s working status,
BidiraI K et al, 2021 [55]	CS	24 to 59 months	569	Ethiopia	Africa	Local	50.8	Educational status of caregiver, marital status of the caregiver, vaccination status of the child, family size	Sex of a child, ARI in the last 2 weeks, dietary diversity, source of drinking water
Kumari T et al, 2024 [125]	CS	6 to 59 months	550	India	Asia	Local	58	Types of family, family size, religion, educational status of father, occupation of father, occupation of mother, washing hands, monthly income, socioeconomic status, age of children, gender, birth spacing between children, antenatal check-up, mode of delivery, place of delivery, immunization, mothers age at pregnancy, ordinal position, sanitation facility, safe drinking water, first feed, initiation of breast feeding, worm infestation, complementary feeding	Educational status of mother
Talapalliwar MR et al, 2014 [126]	CS	0 to 59 months	540	India	Asia	Local	76.3	Age of the child, age at first pregnancy, colostrum received, calorie deficit diet	Presence of any morbidity, child’s anemia level
Berra WG, 2020 [56]	CS	0 to 59 months	525	Ethiopia	Africa	Local	21.3	Household food access category, mothers’ usual feeding practices, mothers attended formal education, child health condition,	Child age, mothers’ report of paid work status
Berra WG et al, 2020 [57]	CS	6 to 23 months	525	Ethiopia	Africa	Local	21.3	Mothers report of no paid work; child health (sick children), inappropriate feeding practices	Age of the child, sex of a child, maternal education
Shahid M et al, 2022 (a) [65]	CS	0 to 59 months	517	Pakistan	Asia	Local	60.8	Birth order, decision to spend women’s earnings, region, mother’s working status, household size, source of drinking water	Sex, age, mother’s education level, mother’s body mass index, wealth index
Permatasari TAE et al, 2023 [69]	CS	0 to 59 months	460	Indonesia	Asia	Local	62	Mother’s age, mother’s education level, father’s education level, father’s occupation, family income, number of children, maternal and child health care, nutritional knowledge, child’s age, child’s sex, child’s birth weight, child’s immunization history, early initiation of breastfeeding, early complementary feeding, parenting style in feeding, toilet facility, defecation habit	Mother’s occupation, balanced nutrition practice, source of drinking water
Gebretsadik MT et al, 2023 [58]	CS	6 to 23 months	438	Ethiopia	Africa	Local	57.3	Birth order	Sex of the child, education, family size, minimum acceptable diet, food security, comorbidity
Sabu KU et al, 2020 [127]	CS	24 to 59 months	314	India	Asia	Local	53.5	Maternal age at marriage, consumption of any fruits or vegetables, frequency food consumption, household land ownership, tribal communities	Food security, birth weight, toilet facilities at home, maternal education, work status, total number of household members
Mohandas A et al, 2023 [34]	CS	0 to 59 months	310	India	Asia	Local	39.7	Initiation of complementary feeding, father’s occupation, any neonatal admission, father’s education	Age of the child, sex of the child, exclusive breastfeeding, birth weight, mother’s occupation, dietary diversity
How ETC et al, 2020 [79]	CS	6 to 59 months	300	Malaysia	East Asia & Pacific	Local	42.3	Smoking status, deworming status, education qualification of father, occupation of mother, income, type of sanitation facility, birth weight, frequency of illness, maternal education	Source of water supply
Indris A et al, 2021 [59]	CS	6 to 23 months	245	Ethiopia	Africa	Local	38.8	Child health condition, mothers report of paid work status, feeding practice	Age of the child
Manjula M et al, 2017 [128]	CS	0 to 59 months	182	India	Asia	Local	47.5	Education of father, overcrowding, age of mother at childbirth, mother’s morbidity during ANC	Family size, education of mother
Jana D et al, 2024 [129]	CS	6 to 59 months	164	India	Asia	Local	68.9	Mother education, education of father, per capita income, birth weight of the participants, mothers’ weight, mothers’ heigh	History of ARI
Roy K et al, 2018 [130]	CS	0 to 59 months	144	India	Asia	Local	36.1	Birth order, hand washing practice, socio-economic status, religion, gender, mother education	Exclusive breastfeeding, birth weight
Workie DL et al, 2021 [24]	CS	0 to 59 months	9411	Ethiopia	Africa	Local	46.7	Region, religion, education level of women and husband, number of under five children, mothers body mass index, multiple birth, birth order, source of drinking water, age of mother at first birth, number of under five children	Place of residence, sex of child, diarrhea, fever, child’s anemia level
Seboka BT et al, 2021 [60]	CS	0 to 59 months	4364	Ethiopia	Africa	Local	46.2	Number of children under five in the household, maternal educational level, paternal educational level, region	Child’s age, birthweight, initiation of breast feeding, mother’s BMI, residence
Fenta HM et al, 2021 [27]	CS	0 to 59 months	29592	Ethiopia	Africa	Local	53.8	Sex of a child, age of the child, vitamin A received, birth order, size of child, type of birth, maternal age, maternal education, paternal education, mothers’ autonomy, maternal employment, mother’s BMI, sanitation facility, number of under-five children, media exposure, wealth index, survey year	Place of residence, comorbidity, dietary diversity, currently breast feeding, sources of water
Vanderhout SM et al, 2020 [131]	CS	6 to 59 months	107,639	India	Asia	Local	56.2	Age of the child, dietary score, wealth quintile, maternal education, mother’s BMI, birth weight, birth size, time of breastfeeding initiation after birth, current breastfeeding, fever or cough in past 2 weeks, home air quality related to cooking fuels used, access to an improved sanitary facility and drinking water source, unsafe disposal of stools, child vaccination status, vitamin A supplementation in the past 6 months, region of residence	Milk consumption
Salazar Burgos et al, 2024 [75]	CS	0 to 59 months	5473	Argentina	South America	National	11.2	NR	Sex, wealth quintiles
Wubetie BY et al, 2024 [61]	CS	0 to 59 months	400	Ethiopia	Africa	Local	49	NR	Disease experience (diarrhea, fever, chronic disease), food security, early initiation of breastfeeding, minimum child dietary diversity score, minimum maternal dietary diversity score, place of birth, child vaccination, maternal empowerment, household wealth, and nutrition-sensitive agricultural practices, household environment (toilet access, kitchen house)
Stiller CK et al, 2020 [132]	CS	6 to 47 months	306	India	Asia	Local	61.6	NR	Age of the child
Shafiq A et al, 2019 [66]	CS	0 to 59 months	3071	Pakistan	Asia	National	52.2	NR	Mother’s education, mothers’ employment, household size, decision-making about visits to family or relatives, wealth index, number of children in house
Savanur MS et al, 2015 [133]	CS	24 to 48 months	634	India	Asia	Local	47.8	NR	Age of the child, sex of the child
Bharali N et al, 2019 [134]	CS	0 to 59 months	362	India	Asia	Local	48.6	NR	Sex of the child
Khan and Raza, 2014 [135]	CS	0 to 59 months	27799	India	Asia	National	60.2	NR	Age, birth-order, duration of breastfeeding, birth-interval, delivery of the child in hospital, wealth index, mother’s BMI, mother’s education, incidence of fever and diarrhea, number of household members
Anisadiyah A et al, 2022 [70]	CS	24 to 59 months	141	Indonesia	Asia	Local	36.2	NR	Exclusive breastfeeding, energy intake, vegetable protein consumption habits
Permatasari TAE et al, 2022 [21]	CS	0 to 59 months	330	Indonesia	Asia	Local	42.1	NR	Maternal height, age of the child, history of infectious diseases, early initiation of breastfeeding, frequency of energy source consumption, frequency of protein source consumption
Al-Sadeeq AH et al, 2018 [81]	CS	6 to 59 months	1292	Yemen	Middle East	Local	70.1	NR	Sex of the child
Asif MA et al, 2019 [67]	CS	0 to 59 months	1870	Pakistan	Asia	National	50.1	NR	Gender, region, wealth status, sex of household head
Bejarano IF et al, 2014 [76]	CS	12 to 59 months	8094	Argentina	South America	Local	4.8	NR	Geographic altitude, sex of the child
SoniI A et al, 2021 [136]	Cohort	0 to 59 months	7868	India	Asia	Local	57.3	NR	Place of residence, monthly spending, highest male education, highest female education, caste, age in years at school enrollment, school type, years of schooling
Mandal GC et al, 2009 [53]	CS	24 to 59 months	894	India	Asia	Local	72.8	NR	Age of the child

NR: Not reported**;CS: Cross-Sectional Study.**

In terms of child age groups, 35 studies (62.5%) included children aged 0–59 months, representing the full under-five population. The remaining studies focused on specific age ranges, including 0–36 months (n = 3), 6–59 months (n = 5), 24–59 months (n = 6), 12–59 months (n = 1), 24–48 months (n = 1), 6–47 months (n = 1), 6–23 months (n = 3), 0–23 months (n = 1), and 2–23 months (n = 1). Among the studies included, the majority were a local geographic focus (62.5%, n = 35) (Table 1). Regarding methodological quality, based on the modified Newcastle-Ottawa Scale (NOS), 56 of the 40 studies (71.4%) were rated as high quality (NOS score 9–10), while 16 studies (28.6%) were classified as good quality (NOS score 7–8) (S2 Table).

### Factors associated with composite index of anthropometric failure (CIAF)

**Fig 2:**
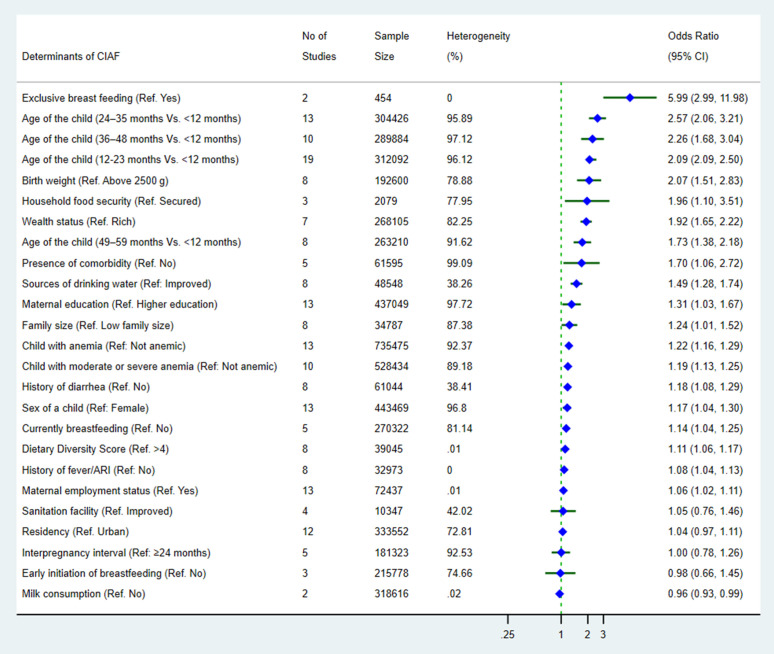
Summary of potential determinants of CIAF among under-five children.

Data from 42 studies were included in the meta-analysis, with confounder-adjusted odds ratios (ORs) used to estimate the pooled effect sizes. Table 2 and Fig 2 summarizes the intrapersonal, interpersonal, and community-level determinants of CIAF among children under five, as identified across the included studies. These factors were categorized using an adapted version of the socio-ecological framework, as described the method section. The statistical adjustments for potential confounders and covariates varied among the studies. The most frequently adjusted factors were maternal education level (n = 16 studies), birth order (n = 13), maternal age (n = 13), wealth index (n = 12), source of drinking water (n = 8), place of delivery (n = 8), mode of delivery (n = 7), maternal BMI (n = 7), sex of the child (n = 9), and toilet facility (n = 6) (Table 1).

**Table 2:** Factors associated with CIAF among children under five in studies included in the meta-analysis, 2000–2024.

Socio-ecological framework	Level of determinants	Factors associated with CIAF	Pooled OR (95% CI)	No of studies included	Sample size	Heterogeneity within the studies		Publication bias (Egger test, p-value)	Evidence of a significant association
						I^2^ (%)	p-value**		
***Intrapersonal level***	Child-individual factors	Sex of a child (Ref: Female)	1.17 (1.04-1.30)	13	443,469	96.80	0.001	0.2447	**⊕**
		Age of the child (12–23 months Vs. < 12 months)	2.09 (1.74-2.50)	19	312,092	96.12	p < 0.001	0.0897	**⊕**
		Age of the child (24–35 months Vs. < 12 months)	2.57 (2.06-3.21)	13	304,426	95.89	p < 0.001	0.0386	**⊕**
		Age of the child (36–48 months Vs. < 12 months)	2.26 (1.68-3.04)	10	289,884	97.12	p < 0.001	0.0013	**⊕**
		Age of the child (49–59 months Vs. < 12 months)	1.73 (1.38-2.18)	8	263,210	91.62	p < 0.001	0.4386	**⊕**
		Presence of comorbidity (Ref. No)	1.70 (1.06-2.72)	5	61,595	99.09	0.03	0.0213	**⊕**
		History of fever/ARI (Ref: No)	1.08 (1.04-1.13)	8	32,973	0.00	0.06	0.0030	**⊕**
		History of diarrhea (Ref. No)	1.18 (1.08-1.29)	8	61,044	38.41	0.16	0.1541	**⊕**
		Child with anemia (Ref: Not anemic) ^#^	1.22 (1.16-1.29)	13	735,475	92.37	p < 0.001	0.0234	**⊕**
		Child with moderate or severe anemia (Ref: Not anemic)	1.19 (1.13-1.25)	10	528,434	89.18	p < 0.001	0.4914	**⊕**
		Birth weight (Ref. Above 2500 g)	2.07 (1.51-2.83)	8	192,600	78.88	p < 0.001	0.2441	**⊕**
		Currently breastfeeding (Ref. No)	1.14 (1.04-1.25)	5	270,322	81.14	p < 0.001	0.0039	**⊕**
		Exclusive breast feeding (Ref. Yes)	5.99 (2.99-11.98)	2	454	0.00	0.52	NA	**⊕**
		Breastfeeding (Ref. No)	1.17 (1.11-1.24)	2	212,408	0.00	0.511	NA	**⊕**
		Early initiation of breastfeeding (Ref. No)	0.98 (0.66-1.45)	3	215,778	74.66	0.05	0.8122	**∅**
	Diet-related factors	Milk consumption (Ref. No)	0.96 (0.93-0.99)	2	318,616	0.02	1.00	NA	**⊗**
		Dietary diversity score (Ref. > 4)	1.11 (1.06-1.17)	8	39,045	0.01	0.03	0.0007	**⊕**
***Interpersonal level***	Maternal factors	Maternal education (Ref. Higher education)	1.31 (1.03-1.67)	13	437,049	97.72	p < 0.001	0.0009	**⊕**
		Maternal employment status (Ref. Yes)	1.06 (1.02-1.11)	13	72,437	0.01	0.03	0.0550	**⊕**
		Interpregnancy interval (Ref: ≥ 24 months)	1.00 (0.78-1.26)	5	181,323	92.53	p < 0.001	0.9886	**∅**
	Household factors	Household food security (Ref. Secured)	1.96 (1.10-3.51)	3	2,079	77.95	0.01	0.6905	**⊕**
		Family size (Ref. Low family size) *	1.24 (1.01-1.52)	8	34,787	87.38	p < 0.001	0.3917	**⊕**
		Wealth status (Ref. Rich)	1.92 (1.65-2.22)	7	268,105	82.25	p < 0.001	0.7273	**⊕**
***Community-***levels	Contextual and structural factors	Residency (Ref. Urban)	1.04 (0.97-1.11)	12	333,552	72.81	p < 0.001	0.0422	**∅**
	Environmental conditions	Sources of drinking water (Ref: Improved)	1.49 (1.28-1.74)	8	48,548	38.26	0.19	0.1760	**⊕**
		Sanitation facility (Ref. Improved)	1.05 (0.76-1.46)	4	10347	42.02	0.21	0.4602	**∅**

*A large family size refers to households with four or more household members; **Cochrane chi-squared test p-value; **⊕**(Red Color) indicates positive association (significantly increased odds of CIAF/ Risk factor), ∅ (Yellow Color) indicates neutral and **⊗**(Green Color) indicates negative association (significantly decreased odds of CIAF); ^#^Hemoglobin level of less than 11 grams/decilitres (g/dl) for children aged 6–59 months old was categorized as anaemic.

### Intrapersonal level determinants

#### Association between sex of a child and CIAF.

**Fig 3:**
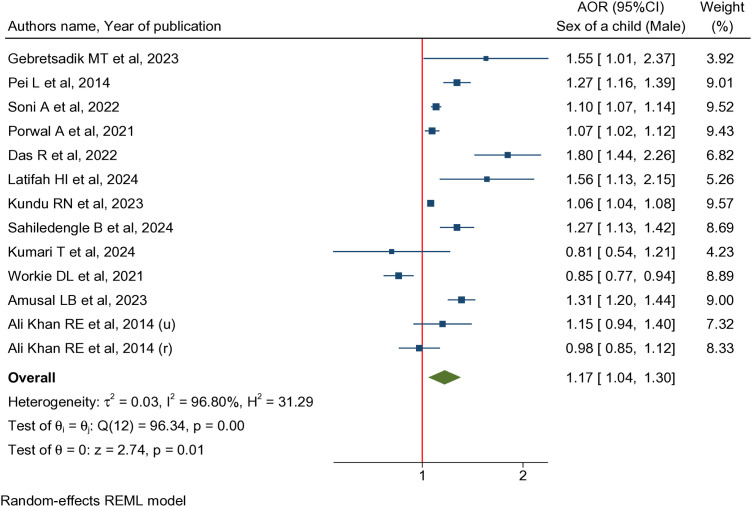
Forest plot showing the association between child’s sex and CIAF.

The pooled adjusted odds ratio (OR) from 13 studies involving 443,469 children under five demonstrated that male children were 17% more likely (pooled OR: 1.17, 95% CI: 1.04-1.30, I^2^ = 96.80%) to have CIAF compared to their female counterparts (Fig 3).

#### The associations between the age of the child and CIAF.

**Fig 4:**
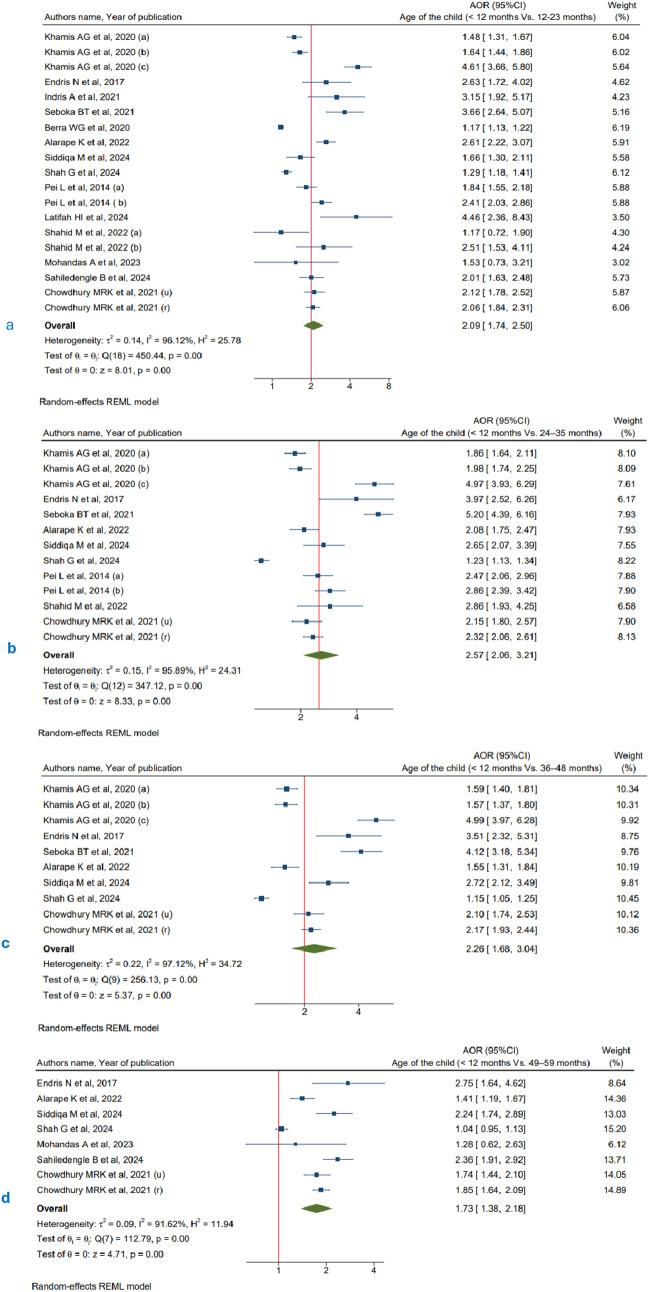
Forest plots illustrating the association between child’s age and CIAF: (a) 12–23 months vs. < 12 months, (b) 24–35 months vs. < 12 months, (c) 36–48 months vs. < 12 months, (d) 49–59 months vs. < 12 months.

The age of children varied across the included primary studies. However, most studies reported that the risk of CIAF increased with age, suggesting that older children were more likely to experience aggregate anthropometric failure compared to infants under 12 months of age. Children aged 12–23 months (pooled OR: 2.09, 95% CI: 1.74-2.50, I² = 96.12%, n = 312,092) and 24–35 months (pooled OR: 2.57, 95% CI: 2.06-3.21, I² = 95.89%, n = 304,426) were 2.09 and 2.57 times higher odds of developing aggregate nutritional failure compared to those under 12 months, respectively. Similarly, children aged 36–48 months are more than twice as likely to experience CIAF (pooled OR: 2.26, 95% CI: 1.68-3.04, I² = 97.12%, n = 289,884). For children aged 49–59 months, the likelihood of CIAF is 1.73 times higher compared to those under 12 months (pooled OR: 1.73, 95% CI: 1.38-2.18, I² = 91.62%, n = 263,210) (Fig 4).

**Fig 5:**
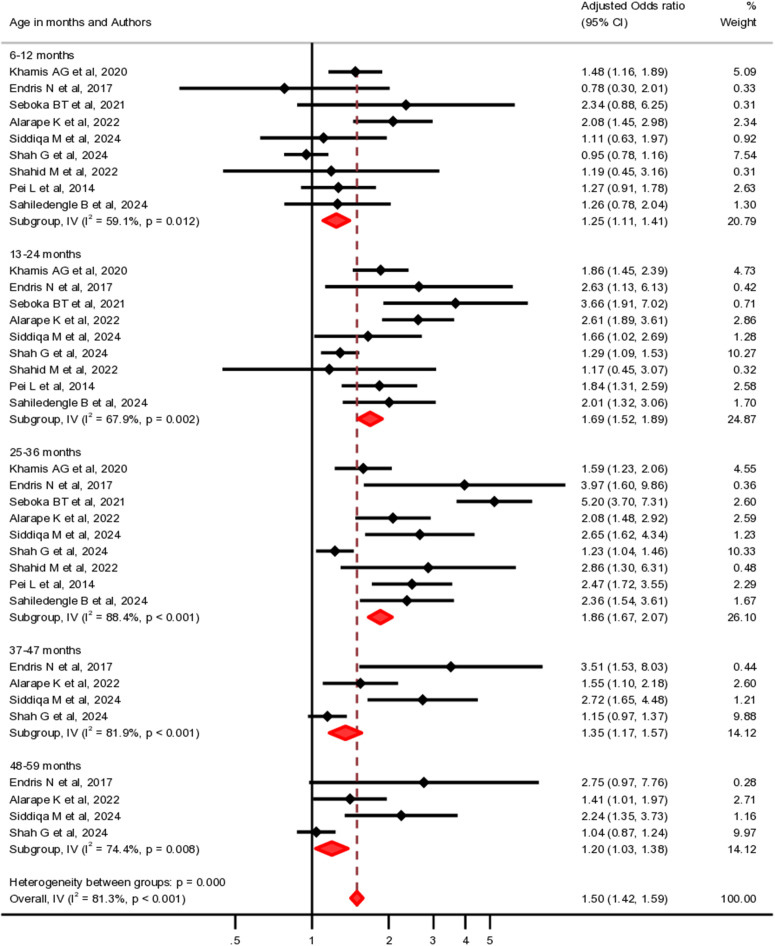
Forest plot showing the associations between child age and CIAF.

To assess the overall association between age and CIAF, we combined all age groups into a single category to estimate the pooled odds for all children under five, using those under 6 months as the reference group. As shown in Fig 5, the pooled analysis revealed a consistent trend across studies, indicating that older children were more likely to experience CIAF (pooled OR: 1.50, 95% CI: 1.42–1.59, I² = 81.3%, n = 275,930),

#### Association between anaemia and CIAF.

Children with anaemia (compared to those who are nonanemic) have 22% higher odds of experiencing the CIAF (pooled OR: 1.22, 95%CI: 1.16-1.29; I² = 92.37%, n = 735,475). Fig A in S1 Fig presents the forest plot on the association between anaemia and CIAF in children. Similarly, children with moderate or severe anaemia have 19% higher odds of CIAF (pooled OR: 1.19, 95%CI: 1.13-1.25; I² = 89.18%, n = 528,434) (Table 2, Fig B in S1 Fig).

#### Childhood illness, comorbidity and CIAF.

Children suffering from diarrhea (pooled OR: 1.18, 95% CI: 1.08-1.29, n = 61,044) and fever (pooled OR: 1.08, 95% CI: 1.04–1.13, n = 32,973) had significantly higher odds of developing CIAF compared to those without these conditions. Similarly, the odds of CIAF were 1.70 times higher among children having comorbid illness than their counterparts (pooled OR: 1.70, 95% CI: 1.06-2.72, n = 61,595) (Table 2). Fig C-E in S1 Fig presents the forest plot on the association between children having a history of diarrhea, fever/ARI and comorbidity and CIAF.

#### Association between birthweight and CIAF.

**Fig 6:**
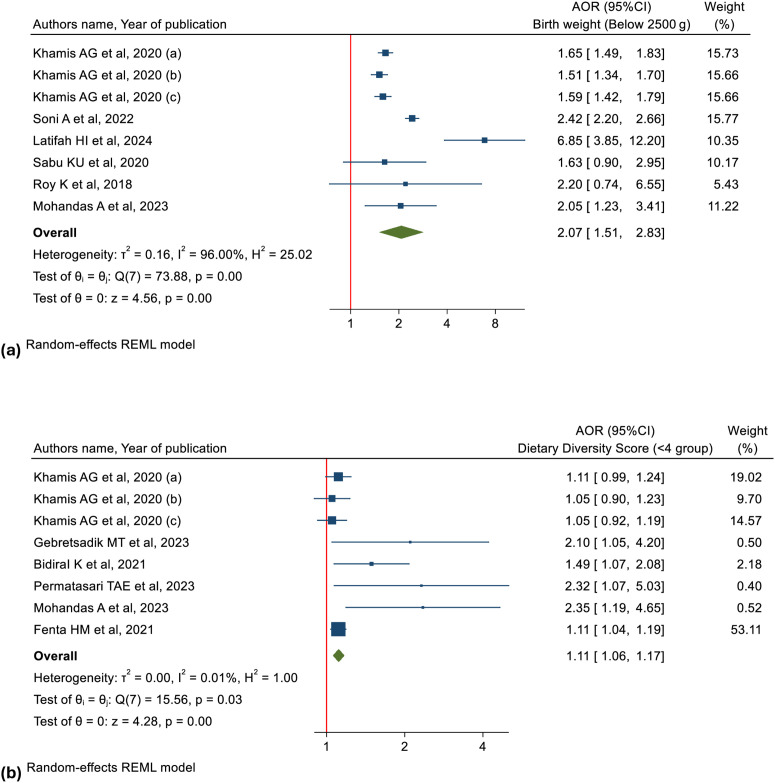
Forest plots illustrating the associations between (a, top) low birth weight and CIAF, and (b, bottom) dietary diversity score and CIAF.

Low-birth weight children were more than twice as likely to experience CIAF compared to those with normal birth weight children (pooled OR: 2.07, 95%CI: 1.51-2.83, I² = 78.88%, n = 192,600). Fig 6 (a) presents the association between birth weight and CIAF among children under five.

#### Association between dietary diversity score, milk consumption, breastfeeding and CIAF.

Children with a lower dietary diversity score were 11% more likely to experience CIAF compared to those with a higher dietary diversity score (pooled OR: 1.11, 95% CI: 1.06–1.17, I² = 0.01%, n = 39,045) (Fig 6(b)). The meta-analysis from two studies involving a total of 318,616 children showed that, children who consume milk had inverse correlation with CIAF (pooled OR: 0.96, 95% CI: 0.93–0.99, I² = 0.02%) (Fig F in S1 Fig). The meta-analysis showed that non-breastfed children had significantly higher odds of anthropometric failure compared to breastfed children (pooled OR = 1.17, 95% CI: 1.11–1.24, I² = 0.0%, n = 212,408) (Fig G in S1 Fig).

### Interpersonal level factors

#### Maternal education, employments status and CIAF.

**Fig 7:**
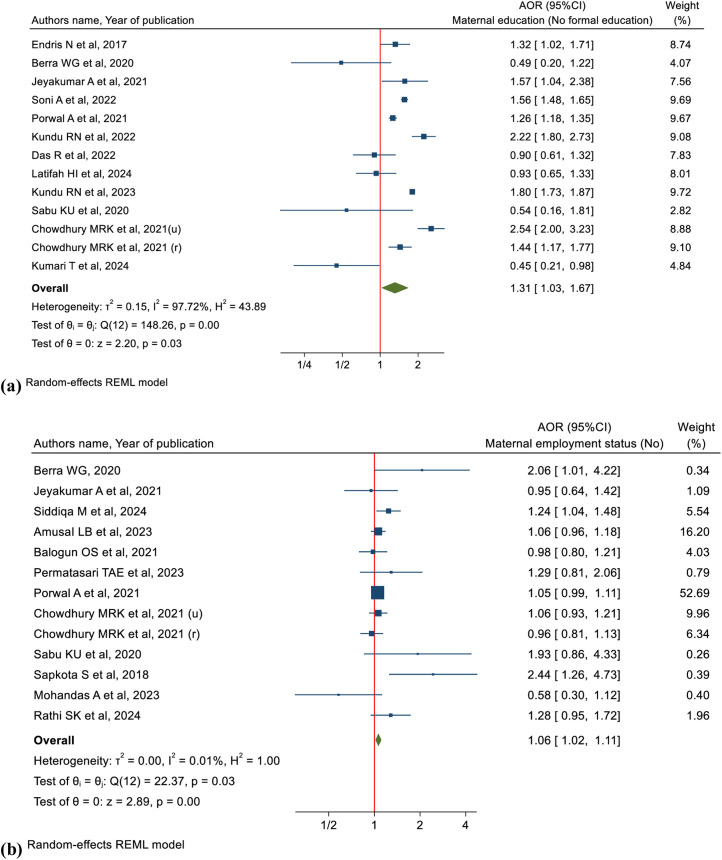
Forest plots illustrate the associations between (a, top) maternal education and CIAF, and (b, bottom) maternal employment status and CIAF.

Children whose mothers have no education were 31% more likely to experience CIAF compared to those whose mothers have higher education (pooled OR: 1.31, 95%CI: 1.03-1.67, I² = 97.72%, n = 437,049) (Fig 7 (a)). Children whose mothers are not employed have a slightly higher likelihood of experiencing CIAF compared to those whose mothers are employed (pooled OR: 1.06, 95%CI: 1.02-1.11, I² = 0.01%, n = 72,437) (Fig 7 (b)).

#### Association between wealth status and CIAF.

**Fig 8:**
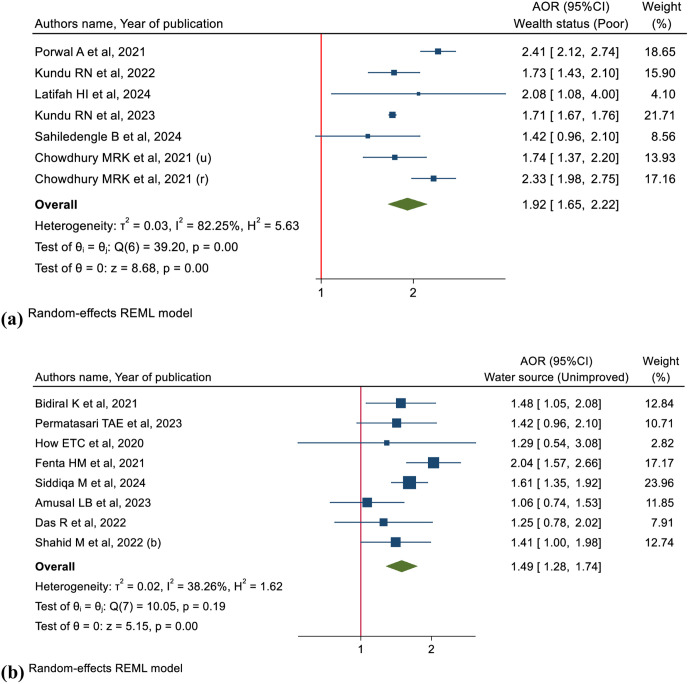
Forest plots illustrate the associations between (a, top) wealth status and CIAF, and (b, bottom) sources of drinking water and CIAF.

Children from richest wealth quintiles as a reference category, the odds of CIAF were 92% higher for children of households with the poorest wealth quintiles (pooled OR: 1.92, 95% CI: 1.65–2.22, I² = 82.25%, n = 268,105) (Fig 8 (a)).

#### Household family size and CIAS.

Children from larger families are more likely to experience CIAF compared to those from smaller families (pooled OR: 1.24, 95%: CI: 1.01-1.52, I² = 87.38%, n = 34,787). Fig H in S1 Fig shows the association between family size and CIAF among under-five children.

#### Associations between household food security with CIAF among under-five children.

The odds of CIAF were nearly twice higher among children from households with insecure food compared to those from food-secure households (pooled AOR: 1.96, 95% CI: 1.10-3.51, I² = 77.95%, n = 2,079). Fig G in S1 Fig presents the associations between household food security and CIAF among under-five children.

### Community-level factors

#### Association between place of residency and CIAF.

Children from rural areas have slightly higher odds of CIAF compared to those from urban areas (pooled AOR: 1.04, 95% CI: 0.97-1.11, I² = 72.81%, n = 333,552); however, our finding is not statistically significant (Fig J in S1 Fig).

#### Sources of drinking water, sanitation facility and CIAF.

Eight studies examined the relationship between sources of drinking water and childhood undernutrition according to CIAF. Meta-analysis result showed that children from households that used unimproved water sources had significantly and 49% higher odds of experiencing CIAF (pooled OR: 1.49, 95% CI: 1.28-1.74, I² = 38.26%, n = 48,548), than those who used improved water sources (Fig 8 (b)). The analysis of sanitation facility type (reference: improved) revealed that the odds of CIAF were slightly higher for children with access to unimproved sanitation facilities, but this association was not statistically significant (pooled OR: 1.05, 95% CI: 0.76–1.46, I² = 42.02%, n = 10,347) (Fig K in S1 Fig).

**Fig 9:**
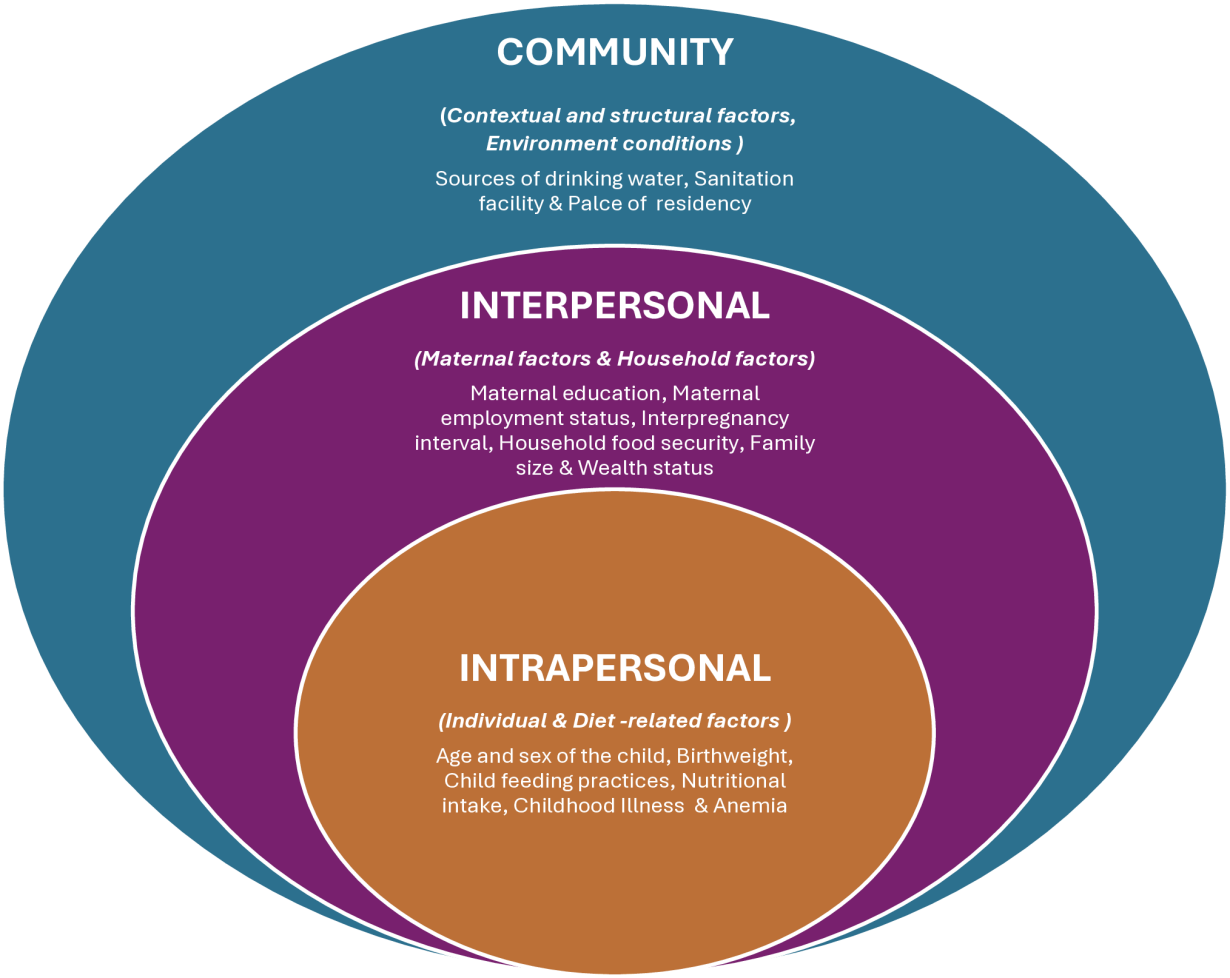
Modified socio-ecological model depicting the multilevel factors associated with childhood undernutrition.

A modified socio-ecological model illustrating the multilevel determinants of the CIAF, encompassing individual, household, and broader community level factors that contribute to childhood undernutrition is presented in Fig 9.

#### Sub-group analysis.

Table 3 presents the results of the subgroup analyses conducted to explore the sources of heterogeneity in the associations between various determinants and CIAF. Given the substantial heterogeneity observed among the included studies, subgroup analyses were performed based on key study characteristics, including child’s age, sample size, study scope, and geographic region, for each identified determinant. Among studies that assessed the association between gender and CIAF, the subgroup analysis based on the age of the children revealed that male children in the age groups of 0–23 months (pooled OR: 1.69, 95%CI: 1.43-2.00) and 0–36 months (pooled OR: 1.27, 95%CI: 1.16-1.39) were found to have significantly higher odds of being affected by CIAF (pooled OR: 1.17, 95% CI: 1.04-1.30). Subgroup analysis by geographical region showed that male children had higher odds of experiencing CIAF compared to females across all regions; however, these findings were not statistically significant.

**Table 3:** Sub-group analysis of factors contributing to high heterogeneity.

Factors	Category	Sub-groups	No of studies included	Pooled OR (95% CI)	Heterogeneity within the studies	
**I**^**2**^ **(%)**	**p-value**
Sex of a child	Age of child	0-23 months	3	1.69 (1.43-2.00)	0.00	0.71
		0-36 months	1	1.27 (1.16-1.39)	–	–
		0-59 months	9	1.08 (0.98-1.18)	95.51	p < 0.001
	Region	Asia	7	1.17 (0.98-1.40)	95.88	p < 0.001
		Africa	4	1.18 (0.92-1.51)	93.27	p < 0.001
		East Asia & Pacific	2	1.15 (0.97-1.38)	93.20	p < 0.001
	Sample size	≤ 10,000	8	1.19 (0.97-1.45)	89.44	p < 0.001
		>10,000	5	1.15 (1.05-1.25)	95.18	p < 0.001
	Scope	Local area focus	5	1.37 (1.07-1.75)	78.55	p < 0.001
		National focus	8	1.09 (0.99-1.20)	95.94	p < 0.001
Wealth index	Age of child	0-23 months	1	2.08 (1.08-4.00)	–	–
		0-59 months	6	1.91 (1.63-2.23)	85.50	0.001
	Region	Asia	5	1.96 (1.68-2.30)	86.81	p < 0.001
		Africa	1	1.42 (0.96-2.10)	–	–
		East Asia & Pacific	1	2.08 (1.08-4.00)	–	–
	Sample size	≤ 10,000	3	1.68 (1.39-2.04)	0.00	0.55
		>10,000	4	2.01 (1.67-2.42)	90.11	0.001
Drinking water sources	Age of child	0-23 months	1	1.25 (0.78-2.02)	–	–
		0-59 months	6	1.51 (1.24-1.83)	51.48	0.10
		24-59 months	1	1.48 (1.05-2.08)	–	–
	Region	Asia	3	1.53 (1.32-1.78)	0.00	0.53
		Africa	3	1.50 (1.03-2.18)	75.48	0.02
		East Asia & Pacific	2	1.40 (0.98-1.99)	0.00	0.84
	Sample size	≤ 10,000	6	1.51 (1.33-1.71)	0.00	0.91
		>10,000	2	1.49 (0.79-2.82)	87.74	p < 0.001
Residency	Age of child	0-23 months	1	0.72 (0.47-1.10)	–	–
		0-59 months	11	1.05 (0.98-1.12)	71.74	p < 0.001
	Region	Asia	5	1.03 (0.99-1.07)	4.02	0.43
		Africa	6	1.09 (0.95-1.24)	83.54	p < 0.001
		East Asia & Pacific	1	0.72 (0.47-1.10)	–	–
	Sample size	≤ 10,000	8	1.01 (0.89-1.12)	72.06	p < 0.001
		>10,000	4	1.08 (0.99-1.17)	78.16	0.01
	Scope	Local area focus	1	0.72 (0.47-1.10)	–	–
		National focus	11	1.05 (0.98-1.12)	71.74	p < 0.001
Morbidity	Sample size	≤ 10,000	3	2.50 (1.53-4.08)	56.16	0.11
		>10,000	2	1.12 (1.02-1.24)	87.99	p < 0.001
	Region	Africa	3	1.74 (0.92-3.30)	87.51	p < 0.001
		Asia	2	1.71 (0.63-4.61)	91.69	p < 0.001
	Age of child	0-23 months	1	3.31 (2.06-5.31)	–	–
		0-59 months	4	1.41 (0.94-2.12)	98.77	p < 0.001
	Scope	Local area focus	3	2.50 (1.53-4.08)	56.16	0.11
		National focus	2	1.12 (1.02-1.24)	87.99	p < 0.001
Maternal education	Age of child	0-23 months	3	0.87 (0.68-1.12)	0.00	0.43
		0-59 months	9	1.52 (1.22-1.88)	97.13	p < 0.001
	Region	Asia	10	1.43 (1.10-1.86)	98.08	p < 0.001
		Africa	2	0.89 (0.34-2.30)	76.33	0.04
		East Asia & Pacific	1	0.93 (0.65-1.33)	–	–
	Sample size	≤ 10,000	8	1.03 (0.68-1.57)	86.98	p < 0.001
		>10,000	5	1.61 (1.34-1.93)	96.73	p < 0.001
Family size	Age of child	0-23 months	1	1.67 (1.06-2.63)	–	–
		0-59 months	6	1.16 (0.93-1.45)	89.89	p < 0.001
		24-59 months	1	1.89 (0.93-3.84)	–	–
	Region	Asia	7	1.20 (0.97-1.48)	88.48	p < 0.001
		Africa	1	1.67 (1.06-2.63)	–	–
	Sample size	≤ 10,000	5	1.34 (1.03-1.74)	45.31	0.15
		>10,000	3	1.13 (0.81-1.59)	96.01	p < 0.001
Birthweight	Region	Asia	4	2.33 (2.00-2.70)	7.91	0.56
		Africa	3	1.59 (1.49-1.69)	0.02	0.53
		East Asia & Pacific	1	6.85 (3.85-12.20)	–	–
	Age of child	0-23 months	1	6.85 (3.85-12.20)	–	–
		0-59 months	7	1.78 (1.50-2.12)	85.77	p < 0.001
	Sample size	≤ 10,000	7	2.03 (1.40-2.95)	95.71	p < 0.001
		>10,000	1	2.42 (2.20-2.66)	–	–
Currently breastfeeding	Region	Asia	1	1.17 (1.11-1.24)	–	–
		Africa	3	1.17 (1.03-1.32)	80.31	p < 0.001
		East Asia & Pacific	1	0.94 (0.76-1.17)	–	–
Age of the child (24–35 months Vs. < 12 months)	Region	Asia	5	2.11 (1.56-2.85)	94.44	p < 0.001
		Africa	6	3.00 (2.02-4.45)	96.71	p < 0.001
		East Asia & Pacific	2	2.66 (2.30-3.07)	21.97	0.26
	Sample size	≤ 10,000	8	2.94 (2.19-3.95)	94.45	p < 0.001
		>10,000	5	2.10 (1.57-2.80)	95.54	p < 0.001
Age of the child (36–48 months Vs. < 12 months)	Region	Africa	6	2.52 (1.63-3.92)	96.96	p < 0.001
		Asia	4	1.92 (1.34-2.77)	96.23	p < 0.001
	Sample size	≤ 10,000	7	2.66 (1.88-3.76)	95.33	p < 0.001
		>10,000	3	1.57 (1.09-2.26)	96.33	p < 0.001
Age of the child (49–59 months Vs. < 12 months)	Region	Africa	3	2.01 (1.34-3.02)	86.78	p < 0.001
		Asia	5	1.60 (1.19-2.14)	82.79	p < 0.001
	Sample size	≤ 10,000	5	2.08 (1.74-2.49)	45.36	0.09
		>10,000	3	1.39 (1.00-1.94)	95.56	p < 0.001
Anemia	Focus	National	12	1.22 (1.16-1.28)	92.85	p < 0.001
		Local	1	3.57 (1.38-9.25)	–	–
	Region	Africa	1	1.44 (1.27-1.64)	–	–
		Asia	12	1.21 (1.15-1.27)	91.98	p < 0.001
	Sample size	≤ 10,000	2	2.00 (0.85-4.69)	70.88	0.06
		>10,000	10	1.20 (1.15-1.27)	92.57	p < 0.001

Subgroup analysis by geographic region for low birthweight demonstrated a consistent and significant association with increased odds of CIAF. The pooled ORs were 2.33 (95% CI: 2.00–2.70) in Asia, 1.59 (95% CI: 1.49–1.69) in Africa, and notably higher in East Asia and the Pacific, with a pooled OR of 6.85 (95% CI: 3.85–12.20) reported from a single study. For maternal education, subgroup analysis showed a significant association between low maternal education and higher odds of CIAF only in studies conducted in Africa (pooled OR: 1.43, 95% CI: 1.10–1.86), whereas studies from other regions did not find statistically significant associations.

Additionally, the subgroup analysis for childhood anaemia demonstrated an elevated risk of CIAF among anaemic children, with regional variation. In Africa, the pooled OR was 1.44 (95% CI: 1.27–1.64), indicating a moderately higher risk compared to Asia, where the pooled OR was 1.21 (95% CI: 1.15–1.27). Subgroup analysis by region showed that children from poorer households had significantly higher odds of experiencing CIAF across most regions. In Asia, the association was strong and statistically significant (pooled OR: 1.96, 95% CI: 1.68–2.30).

#### Publication bias.

**Fig 10:**
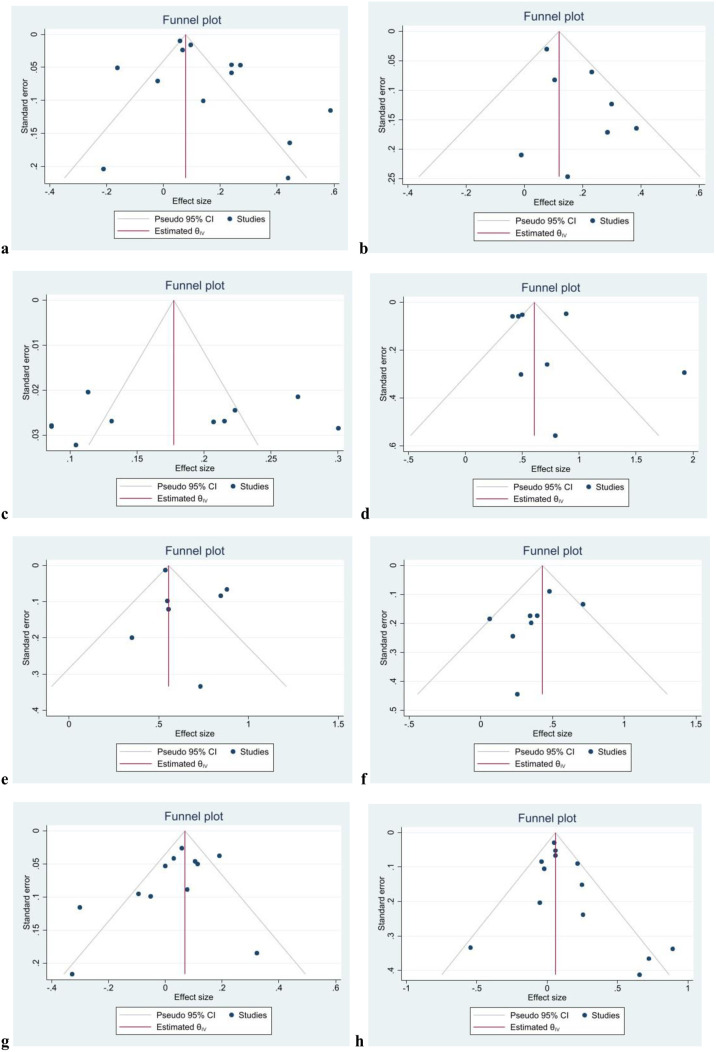
Funnel plots for assessing publication bias across studies examining factors associated with CIAF: (a) child’s sex, (b) diarrhea, (c) moderate or severe anemia, (d) birth weight, (e) wealth status, (f) sources of drinking water, (g) place of residency, and (h) maternal employment status.

Table 3 summarizes the publication bias egger’s statistical test p-value results. No publication bias was detected for associations between gender and CIAF (Egger’s test p-value = 0.2447) (Fig 10(a)), history of diarrhea (p = 0.1541) (Fig 10(b)) moderate/severe anemia (p = 0.4914) (Fig 10(c)), birthweight (p = 0.2441) (Fig 10(d)), wealth status (p = 0.7273) (Fig 10(e)), and sources of drinking water (p = 0.1760) (Fig 10(f), place of residency (p = 0.0422) (Fig 10(g)), and maternal employment status (p = 0.0550) (Fig 10(h)). The funnel plot for a symmetry for visual inspection of publication bias were also presented in S2 Fig.

#### Meta-regression analysis.

To further investigate sources of heterogeneity across studies, we conducted meta-regression analyses using sample size and year of publication as moderators. The results revealed several significant patterns. Specifically, among studies examining the relationship between wealth index and CIAF, larger sample sizes were associated with a 12% decrease in the odds of CIAF (p = 0.008). For studies assessing birthweight, each additional year in publication was associated with a 24% increase in the odds of reporting a significant association with CIAF (p < 0.001), suggesting growing recognition of birthweight as a determinant in more recent studies. Additionally, an inverse relationship between sample size and CIAF was also observed in studies on child morbidity (p < 0.001) and anemia (p = 0.006), indicating that studies with larger samples tended to report lower odds of CIAF. S1 Data contains the data used in this systematic review and meta-analysis.

#### Sensitivity analysis.

We have conducted a leave-one-out sensitivity analysis to identify the influence of one study on the overall pooled odds ratio estimate. The overall estimate of the included studies did not appear to be affected by the removal or addition of a single study at a time, suggesting the robustness of our pooled OR estimate. The leave-one-out sensitivity analysis results for different determinant factors were presented S3 Fig.

## Discussion

Undernutrition among children under five remains a significant global public health challenge, with its most severe impact in LMICs. Traditional indicators such as underweight, wasting and stunting do not fully capture the scope and complexity of childhood undernutrition. The composite index of anthropometric failure (CIAF), which aggregates all three types of undernutrition, offers a more comprehensive estimate of the overlapping burden. Despite various epidemiological studies exploring the determinants of CIAF, no global systematic review has synthesized these findings to date. This is the first comprehensive systematic review and meta-analysis that aimed to summarize the existing evidence on the potential determinants of the CIAF among under-five children. A systematic review and meta-analysis provide a robust, unbiased synthesis of evidence, and informing evidence-based practice. Our findings indicate that children with anemia, low birthweight, poor dietary diversity, a history of illness, and older age were more likely to experience CIAF. Maternal and household factors, such as lower maternal education, unemployment, poverty, larger family sizes, and food insecurity, were also linked to higher odds of CIAF. At the community level, access to unimproved drinking water was significantly associated with increased CIAF risk.

### Gender and CIAF

Although females are often perceived as more disadvantaged from a gender-based disparities, our review found that male children have significantly higher odds of experiencing CIAF compared with their female counterparts. This aligns with epidemiological studies from multiple countries [83–86], suggesting male children are more vulnerable to undernutrition due to weaker immune responses, higher nutritional needs, and potential socio-cultural factors [87,88]. A previous systematic review also reported a higher prevalence of undernutrition among male compared to females [89]. However, the underlying biological and socio-cultural mechanisms remain understudied and require further investigation.

### Age and CIAF

Consistent with previous studies [90–92], our meta-analysis found a significant association between child age and the likelihood of experiencing CIAF. Compared to very young children under one year of age, children aged 24–59 months had higher odds of experiencing CIAF. Notably, the odds of CIAF increased with age, peaking at 24–35 months (OR: 2.57, 95% CI: 2.06-3.21), followed by 36–48 months (OR: 2.26, 95% CI: 1.68-3.04) and 49–59 months (OR: 1.73, 95% CI: 1.38-2.18). This age related trend suggests that as children grow older, their risk of anthropometric failure rises, possibly due to dietary shifts, inadequate complementary feeding, limited dietary diversity, higher nutritional demands, repeated exposure to infections, and poor access to nutrition and healthcare services [93–95]. These findings highlight the critical need for targeted nutritional interventions beyond infancy, particularly during early childhood, to mitigate the long-term consequences of undernutrition.

### Anaemia, childhood illnesses and CIAF

While some studies have explored the link between anaemia and undernutrition [96,97], there remains a paucity of research on the association between anaemia and multiple anthropometric failures. In this review, anaemia was significantly associated with CIAF. Children with any form of anaemia had 22% higher odds of experiencing CIAF compared to non-anaemic children. Additionally, the odds of CIAF were 29% higher among children with moderate or severe anaemia than their non-anaemic counterparts. Although the mechanism by which anaemia affects children’s growth is complex, one possible explanation is that iron deficiency anaemia weakens the immune system, making the children more susceptible to infections and childhood illnesses. Persistent infections in early childhood can impair growth by reducing nutrient absorption [98]. A recent systematic review by Oktarina et al. (2024) [99], reported a significant association between iron deficiency anaemia and stunting in children. Another possible mechanism could be the vicious cycle between anaemia and undernutrition, where one condition exacerbates the other [100–102]. These findings highlight the need for further investigation into the role of anaemia in multiple forms of undernutrition and to address both conditions simultaneously. Our review also found that childhood illnesses such as diarrhea and fever were positively associated with CIAF. This finding can be easily explained by the vicious cycle of undernutrition and infection [103]. Childhood illnesses often compromise nutrient absorption and exacerbate poor growth, increasing the risk of multiple anthropometric failures.

### Birthweight and CIAF

The long-term association between low birth weight (LBW) and childhood linear growth failure is well established, with recent systematic review evidence indicating that LBW more than doubles the risk of stunting [104]. Our review similarly found that children with LBW were more than twice as likely to experience CIAF compared to those with normal birth weight. Regional subgroup analysis further confirmed a positive association between LBW and CIAF, with consistent findings across Africa, Asia, and the East Asia and Pacific region. This association may be explained by intrauterine growth restriction due to poor maternal nutrition, which predisposes infants to anthropometric failure. Additionally, LBW children are more susceptible to infections, which further contribute to anthropometric failure by impairing nutrient absorption. Together, these factors underpin the strong association between LBW and multiple forms of childhood undernutrition [104–106].

### Food security and CIAF

There is broad consensus that household food security, including access to adequate and diversified foods, is essential for children’s linear growth and development. Our findings indicate that children from food-insecure households were nearly twice as likely to experience CIAF compared to those from food-secure households. This is supported by a systematic review of 36 studies, which found a significant association between household food insecurity and childhood undernutrition, particularly stunting and underweight [107]. As far as we are aware, there is no study that has examined either the impact or association between household food security on CIAF, making our findings possibly the first in this regard. However, it is important to note that these findings are based on only three studies identified through our systematic search, all conducted in low-income settings. The relationship between household food security and CIAF can be explained by the impact of food insecurity on young children, who often suffer from inadequate dietary intake. This deficiency in dietary intake leads to both acute and chronic malnutrition, increasing the occurrence of multiple nutritional failures. Prolonged food insecurity also negatively affects maternal and fetal health by increasing the risk of fetal growth restriction, which can result in long-term stunting. If stunting is not addressed within the first two years of life, it becomes difficult to reverse, resulting in long-term developmental outcomes [2,108].

Our meta-analysis highlights the crucial role of breastfeeding in influencing children’s undernutrition status. We found that children who are not currently breastfed and those with no history of exclusive breastfeeding have higher odds of experiencing CIAF. Additionally, children with a lower dietary diversity score were more likely to experience CIAF compared to their counterparts. Breastfeeding and adequate dietary intake are well-established as critical factors in supporting child growth and nutritional status [109]. The protective effects of breastfeeding against infections support optimal child growth, while diversified and adequate complementary feeding further helps prevent multiple anthropometric failures [110].

### Wealth status and CIAF

The meta-analysis revealed a strong association between household wealth status and CIAF, with children from the poorest wealth quintile having 92% higher odds of experiencing CIAF compared to those from the richest quintile. This finding is unsurprising, as extensive epidemiological evidence has consistently demonstrated the interconnected relationship between poverty and undernutrition [14,111,112]. Studies have provided compelling evidence of pronounced wealth-related disparities in child undernutrition, with children from lower wealth quintiles facing substantially higher risks of multiple anthropometric failures [113–115]. Poverty significantly affects household food security and food availability, and limits access to nutritious and diverse foods. It also has a profound impact on parental education levels, which in turn influences child-feeding practices and healthcare-seeking behaviors. These findings have important implications, calling for strengthened global commitment to ending extreme poverty, which has significant impacts on child nutrition and overall development.

### Sources of drinking water and CIAF

Our review found that children from households relying on unimproved water sources had 49% higher odds of experiencing CIAF. The link between undernutrition and poor water, sanitation, and hygiene (WASH) conditions has been widely studied and remains a persistent risk factor [116]. Unimproved drinking water sources increase the risk of childhood undernutrition, largely due to increased exposure to microbial contamination and diarrheal diseases. These findings underscore the critical role of access to improved water sources in reducing childhood undernutrition through nutrition-sensitive interventions such as “baby-WASH” programs. These initiatives aim to improve WASH practices, minimize exposure to environmental pathogens, and reduce the incidence of diarrheal diseases, thereby supporting optimal child growth [117,118].

### Household family size and CIAF

Our meta-analysis findings indicated significantly higher odds of CIAF among children from large families. Several factors contribute to this association: (i) Limited access to diversified and adequate food in these households, which often leads to acute malnutrition and, if prolonged, results in chronic and multiple forms of undernutrition; (ii) Food insecurity, which is more prevalent in large households, further increases the risk of undernutrition; and (iii) Economic constraints in large families limit access to nutritious food and essential healthcare, exacerbating poor nutritional outcomes. Consistent with our findings, a recent meta-analysis by Elmighrabi et al [112], found that larger family sizes, whether in terms of the number of family members or higher birth order, were associated with lower nutrition indices. However, there remains a lack of comprehensive research on how family size specifically the age structure and number of children impacts child nutritional status and contributes to multiple anthropometric failures.

### Maternal education and employment and CIAF

Lower maternal education and maternal unemployment were significantly associated with CIAF, whereas rural residence showed no significant association. Several reviews on undernutrition have also highlighted the protective effects of maternal education and employment on child nutritional outcomes [38,112,119]. Our results showed that children of mothers with lower or no education had 31% higher odds of experiencing CIAF compared to those with educated mothers, likely due to limited health knowledge and reduced access to resources. Maternal education significantly influences child-feeding practices and healthcare-seeking behavior, as educated mothers are more likely to make informed decisions regarding nutrition and medical care [120]. Similarly, children of unemployed mothers had slightly higher odds of experiencing CIAF compared to those with employed mothers, potentially due to reduced household income and limited access to nutritious food and healthcare.

## Strengths and limitations

To the best of the authors’ knowledge, this is the first systematic review and meta-analysis to synthesize global evidence on the determinants of child undernutrition using the CIAF among children under five. While this review offers a comprehensive overview, several important limitations worth noting. First, all included studies were cross-sectional, which prevents us from inferring cause-and-effect relationships from the pooled odds ratio estimates. Second, as with any observational research, the included primary studies may have been subject to recall and social desirability biases. Third, nearly all the studies included in this review were conducted in LMICs, limiting the representation from high-income countries and thus reducing the global generalizability of the findings. Fourth, the high heterogeneity among the included studies may have introduced bias in the summary estimates. Fifth, CIAF is a complex issue, and not all potential determinants were covered due to a lack of available data. Despite these limitations, the findings provide valuable insights into the potential determinants of multiple anthropometric failures in children under five in low-income settings.

## Policy implications

Findings from this study will help policymakers and public health professionals identify the most consistent, multi-level determinants of CIAF, guiding targeted actions to reduce child undernutrition particularly in low-income settings. These findings highlight the need for holistic, multisectoral policies that integrate individual, household, and community-level interventions, with a focus on addressing modifiable risk factors to effectively combat child undernutrition. Policies should prioritize modifiable risk factors by promoting maternal education, improving childcare practices, ensuring early treatment of childhood illnesses, enhancing dietary diversity and food adequacy, expanding access to safe water, and strengthening food security. This study also highlights critical research gaps in underrepresented sub-regions, underscoring the need for localized investigations to better understand context-specific drivers of CIAF and inform tailored policy responses.

## Conclusions

Overall, our findings indicate that CIAF is not solely influenced by individual level factors but also by the interplay of various interpersonal and community-level factors. Intrapersonal factors associated with CIAF included demographic and health-related characteristics such as older child age, a history of diarrhea or fever, anaemia, and low birth weight. Poor dietary diversity significantly increased the odds of CIAF, whereas milk consumption was associated with a modest protective effect. At the interpersonal factors such as lower maternal education, unemployment, poverty (living in households with poor wealth quintile), larger family size, and household food insecurity were significantly linked to increased odds of CIAF. At the community level, reliance on unimproved drinking water sources was associated with higher odds of CIAF among children. These findings emphasize the importance of considering multiple, multi-level ecological factors when addressing multiple anthropometric failures in children, particularly modifiable risk factors. Furthermore, existing nutrition policies and intervention strategies, such as the WHO double-duty actions-which adopt a holistic approach to address all forms of malnutrition should be reinforced. It is essential to revitalize both nutrition-specific and nutrition-sensitive interventions through a comprehensive, multisectoral approach that integrates individual, household, and community-level determinants.

## Supporting information

S1 Checklist PRISMA checklist for reporting systematic reviews. (PDF)

S1 Text Detailed search strategies, keywords, and MeSH terms used for each database. (PDF)

S1 Table List of articles excluded after full-text review, including the specific reasons for their exclusion. (PDF)

S2 Table Quality assessment of included studies using the adapted Newcastle-Ottawa Scale for cross-sectional studies. (PDF)

S1 Fig Forest plots illustrating associations between child, household, and environmental factors and CIAF among children under five. (PDF)

S2 Fig Funnel plots assessing publication bias in studies examining factors associated with CIAF among children under five:(a) birth interval, (b–e) child’s age groups, (f) fever, (g) comorbidity, (h) currently breastfeeding, (i) dietary diversity, (j) family size and (k) maternal education. (PDF)

S1 Data Metadata. (XLSX)

S3 Fig A leave-one-out sensitivity analysis results. (PDF)
