# Supplementary material for: Socio-ecological determinants of multiple anthropometric failures among under-five children: A systematic review and meta-analysis of observational studies
Source: PLOS Glob Public Health. 2025 Jul 31;5(7):e0005008. doi: 10.1371/journal.pgph.0005008 (PMC12312983; doi:10.1371/journal.pgph.0005008)
Supplement: S1 Text — (PDF) [file pgph.0005008.s002.pdf]

S1_Text: Detailed search strategies, keywords, and MeSH terms used for each database

Contents

MEDLINE SEARCH STRATEGY 2

CINAHL SEARCH STRATEGY 3

EMBASE SEARCH STRATEGY 4

SCOPUS SEARCH STRATEGY 4

ProQuest SEARCH STRATEGY 5

Global Index Medicus SEARCH STRATEGY 6

Cochrane Library SEARCH STRATEGY 6

MEDLINE SEARCH STRATEGY

#1 ANTHROPOMETRIC FAILURE

KEYWORDS: "Nutritional Failure" OR “Nutrition Disorder” OR “Nutritional Disorder*” OR “Nutritional Deficienc*” OR “Malnourishment*” OR "Protein-Energy Malnutrition" OR “malnourish*” OR “Severe Acute Malnutrition” OR “Child Malnutrition” OR “Malnutrition in Children” OR “nutritional status” OR “Undernutrition” OR “Malnourished Children” OR “stunting*” OR “wasting” OR “underweight” OR “chronic undernutrition” OR “wasting and underweight” OR “underweight and stunting” OR “Nutrition Assessment” OR "anthropometric*" OR "Anthropometric Z-scores" OR "Anthropometric Indices" OR “anthropometric index” OR “anthropometric failure” OR “anthropometric deficits” OR “CIAF” OR “CISAF” OR “ECIAF”

SUBJECT HEADING: Nutrition Disorders [MeSH Terms] OR "Malnutrition" [MeSH Terms] OR "Protein-Energy Malnutrition" [MeSH Terms] OR "Severe Acute Malnutrition" [MeSH Terms] OR “Nutrition Assessment” [MeSH Terms] OR "Anthropometry" [MeSH Terms]

#2 COMPOSITE INDEX

KEYWORDS: "composite index" OR “multiple failures” OR “overall burden” OR “aggregate*” OR "coexist*" OR “co-occurring” OR "concurrent*" OR “concomitant”

SUBJECT HEADING: Syndemic [MeSH Terms]

#3 DETERMINANTS

KEYWORDS: “determinant*” OR “determinant factors” OR “effect” OR “Associated factor*” OR "factor*" OR “association” OR “risk factor*” OR "risk" OR "predictor*" OR “driver*” OR “factors associated” OR “regression analysis” OR “Multivariate Analysis” OR "epidemiology" OR “Prevalence” OR “Prevalences” OR "burden*" OR "Prevalence Stud*" OR “Observational Stud*” OR "Cross Sectional Stud*" OR "Cross Sectional Survey*" OR "Cross Sectional Analyses" OR "Cross Sectional Analysis" OR “Case-Control Stud*” OR “Cohort Stud*” OR “Longitudinal Stud*” OR “Quasi-Experimental Studies” OR “Non-Randomized Controlled Trials” OR “Randomized Controlled Trials”

SUBJECT HEADING: "Risk Factors" [MeSH Terms] OR “Prevalence” [MeSH Terms] OR “Cross-Sectional Studies” [MeSH Terms] OR “Case-Control Studies” [MeSH Terms] OR “Cohort Studies” [MeSH Terms] OR “Longitudinal Studies” [MeSH Terms] OR “Non-Randomized Controlled Trials as Topic” [MeSH Terms] OR “Randomized Controlled Trials as Topic” [MeSH Terms]

#4 Under-five children

KEYWORDS: “Child*” OR “Minor*” OR “under-five child*” OR “under-5 child*” OR "children under 5" OR “Children aged 6-59 months” OR “Preschool” OR “Preschool Child*” OR “Early Childhood” OR “Infants” OR "infant and child" OR “Infants and young children” OR "toddler*" OR "Pre-school age children" OR "early years" OR "young children"

SUBJECT HEADING: "Child"[MeSH Terms] OR “Pediatrics” [MeSH Terms] OR “Child, Preschool” [MeSH Terms] OR “Infant” [MeSH Terms]

#5 Search #1 AND #2

#6 Search #5 AND #3

#7 Search #6 AND #4

CINAHL SEARCH STRATEGY

Search ID#	Search Terms
	ANTHROPOMETRIC FAILURE
S1	AB "Nutritional Failure" OR AB “Nutrition Disorder” OR AB “Nutritional Disorder*” OR AB “Nutritional Deficienc*” OR AB “Malnourishment*” OR AB "Protein-Energy Malnutrition" OR AB “malnourish*” OR AB “Severe Acute Malnutrition” OR AB “Child Malnutrition” OR AB “Malnutrition in Children” OR AB “nutritional status” OR AB “Undernutrition” OR AB “undernourishment” OR AB “Malnourished Children” OR AB “stunting*” OR AB “wasting” OR AB “underweight” OR AB “chronic undernutrition” OR AB (“wasting and underweight”) OR AB (“underweight and stunting”) OR AB “Nutrition Assessment” OR AB "anthropometric*" OR AB "Anthropometric Z-scores" OR AB "Anthropometric Indices" OR AB “anthropometric index” OR AB “anthropometric failure” OR AB “anthropometric deficits” OR AB “CIAF” OR AB “CISAF” OR AB “ECIAF”
S2	*MH "Child Nutrition" OR MH “Child Nutrition Disorders” OR MH “Protein-Energy Malnutrition” OR MH “Malnutrition” OR MH “Undernutrition” OR MH “Anthropometry”*
S3	S1 OR S2
	COMPOSITE INDEX
S4	AB "composite index" OR AB “multiple failures” OR AB “overall burden” OR AB “aggregate failure*” OR AB "coexist*" OR AB “co-occurring” OR AB "concurrent*" OR AB “concomitant” OR AB “Syndemic*”
S5	*MH “Syndemic”*
S6	S4 OR S5
	DETERMINANTS
S7	AB “determinant*” OR AB “determinant factors” OR AB “Associated factor*” OR AB "factor*" OR AB “association” OR AB “risk factor*” OR AB "risk" OR AB "predictor*" OR AB “driver*” OR AB "epidemiology" OR AB “Prevalence” OR AB “Prevalences” OR AB "burden*" OR AB "Prevalence Stud*" OR AB “Observational Stud*” OR AB "Cross Sectional Stud*" OR AB “Case-Control Stud*” OR AB “Cohort Stud*” OR AB “Longitudinal Stud*” OR AB “Quasi-Experimental Studies” OR AB “Non-Randomized Controlled Trials” OR AB “Randomized Controlled Trials”
S8	*MH “Prevalence” OR MH “Cross Sectional Studies” OR MH “Case Control Studies” OR MH “Prospective Studies” OR MH “Randomized Controlled Trials” OR MH “Quasi-Experimental Studies” OR MH “Nonexperimental Studies” OR MH "Risk Factors"*
S9	S7 OR S8
	UNDER-FIVE CHILDREN
S10	AB “Child*” OR AB “Minor*” OR AB “under-five child*” OR AB “under-5 child*” OR AB "children under 5" OR AB “Children aged 6-59 months” OR AB “Preschool” OR AB “Preschool Child*” OR AB “Early Childhood” OR AB “Infants” OR AB ("infant and child") OR AB (“Infants and young children”) OR AB "toddler*" OR AB "Pre-school age children" OR AB "early years" OR AB "young children"
S11	MH "Child" OR MH “Child, Preschool” OR MH “Infant”
S12	S10 OR 11
S13	S3 AND S6
S14	S13 AND S9
S15	S14 AND S12

EMBASE SEARCH STRATEGY

1. ("Nutritional Failure" OR "Nutrition Disorder" OR "Nutritional Disorder*" OR "Nutritional Deficienc*" OR "Malnourishment*" OR "Protein-Energy Malnutrition" OR "malnourish*" OR "Severe Acute Malnutrition" OR "Child Malnutrition" OR "Malnutrition in Children" OR "nutritional status" OR "Undernutrition" OR "Malnourished Children" OR "stunting*" OR "wasting" OR "underweight" OR "chronic undernutrition" OR "wasting and underweight" OR "underweight and stunting" OR "Nutrition Assessment" OR "anthropometric*" OR "Anthropometric Z-scores" OR "Anthropometric Indices" OR "anthropometric index" OR "anthropometric failure" OR "anthropometric deficits" OR "CIAF" OR "CISAF" OR "ECIAF").mp. [mp=title, abstract, heading word, original title, keyword heading word, floating subheading word, candidate term word]
2. ("Composite index" OR "multiple failures" OR "overall burden" OR "aggregate failure*" OR "coexist*" OR "co-occurring" OR "concurrent*" OR "concomitant").mp. [mp=title, abstract, heading word, original title, keyword heading word, floating subheading word, candidate term word]
3. ("determinants" OR "determinant*" OR "determinant factors" OR "effect" OR "Associated factor*" OR "factor*" OR "association" OR "risk factor*" OR "risk" OR "predictor*" OR "driver*" OR "factors associated" OR "regression analysis" OR "Multivariate Analysis" OR "epidemiology" OR "Prevalence" OR "Prevalences" OR "burden*" OR "Prevalence Stud*" OR "Observational Stud*" OR "Cross Sectional Stud*" OR "Cross Sectional Survey*" OR "Cross Sectional Analyses" OR "Cross Sectional Analysis" OR "Case-Control Stud*" OR "Cohort Stud*" OR "Longitudinal Stud*" OR "Quasi-Experimental Studies" OR "Non-Randomized Controlled Trials" OR "Randomized Controlled Trials"). mp. [mp=title, abstract, heading word, original title, keyword heading word, floating subheading word, candidate term word]
4. ("Child*" OR "Minor*" OR "under-five child*" OR "under-5 child*" OR "children under 5" OR "Children aged 6-59 months" OR "Preschool" OR "Preschool Child*" OR "Early Childhood" OR "Infants" OR "infant and child" OR "Infants and young children" OR "toddler*" OR "Pre-school age children" OR "early years" OR "young children"). mp. [mp=title, abstract, heading word, original title, keyword heading word, floating subheading word, candidate term word]
5. Search #1 AND #2
6. Search #5 AND #3
7. Search #6 AND #5

SCOPUS SEARCH STRATEGY

(TITLE-ABS ("Nutritional Failure" OR "Nutrition Disorder" OR "Nutritional Disorder*" OR "Nutritional Deficienc*" OR "Malnourishment*" OR "Protein-Energy Malnutrition" OR "malnourish*" OR "Severe Acute Malnutrition" OR "Child Malnutrition" OR "Malnutrition in Children" OR "nutritional status" OR "Undernutrition" OR "Malnourished Children" OR "stunting*" OR "wasting" OR "underweight" OR "chronic undernutrition" OR "wasting and underweight" OR "underweight and stunting" OR "Nutrition Assessment" OR "anthropometric*" OR "Anthropometric Z-scores" OR "Anthropometric Indices" OR "anthropometric index" OR "anthropometric failure" OR "anthropometric deficits" OR "CIAF" OR "CISAF" OR "ECIAF")) AND (TITLE-ABS-KEY ("composite index" OR "multiple failures" OR "overall burden" OR "aggregate failure*" OR "coexist*" OR "co-occurring" OR "concurrent*" OR "concomitant")) AND ((TITLE-ABS-KEY ("determinants" OR "determinant*" OR "determinant factors" OR "effect" OR "Associated factor*" OR "factor*" OR "association" OR "risk factor*" OR "risk" OR "predictor*" OR "driver*" OR "factors associated" OR "regression analysis" OR "Multivariate Analysis" OR "epidemiology" OR "Prevalence" OR "Prevalences" OR "burden*" OR "Prevalence Stud*" OR "Observational Stud*" OR "Cross Sectional Stud*" OR "Cross Sectional Survey*" OR "Cross Sectional Analyses" OR "Cross Sectional Analysis" OR "Case-Control Stud*" OR "Cohort Stud*" OR "Longitudinal Stud*" OR "Quasi-Experimental Studies" OR "Non-Randomized Controlled Trials" OR "Randomized Controlled Trials"))) AND (TITLE-ABS ("Child*" OR "Minor*" OR "under-five child*" OR "under-5 child*" OR "children under 5" OR "Children aged 6-59 months" OR "Preschool" OR "Preschool Child*" OR "Early Childhood" OR "Infants" OR "infant and child" OR "Infants and young children" OR "toddler*" OR "Pre-school age children" OR "early years" OR "young children")) AND (LIMIT-TO (DOCTYPE , "ar"))

ProQuest SEARCH STRATEGY

S1	Anthropometric failure (title/Abstract)
	abstract("Nutritional Failure" OR "Nutrition Disorder" OR "Nutritional Disorder*" OR "Nutritional Deficienc*" OR "Malnourishment*" OR "Protein-Energy Malnutrition" OR "malnourish*" OR "Severe Acute Malnutrition" OR "Child Malnutrition" OR "Malnutrition in Children" OR "nutritional status" OR "Undernutrition" OR "undernourishment" OR "Malnourished Children" OR "stunting*" OR "wasting" OR "underweight" OR "chronic undernutrition" OR "wasting and underweight" OR "underweight and stunting" OR "Nutrition Assessment" OR "anthropometric*" OR "Anthropometric Z-scores" OR "Anthropometric Indices" OR "anthropometric index" OR "anthropometric failure" OR "anthropometric deficits" OR "CIAF" OR "CISAF" OR "ECIAF")<br>*Additional limits - Source type: Scholarly Journals*
S2	Composite index
	abstract ("composite index" OR "multiple failures" OR "overall burden" OR "aggregate failure*" OR "coexist*" OR "co-occurring" OR "concurrent*" OR "concomitant")<br>*Additional limits - Source type: Scholarly Journals*
S3	Determinants
	abstract("epidemiology" OR "Prevalence" OR "Prevalences" OR "burden*" OR "Prevalence Stud*" OR "Observational Stud*" OR "Cross Sectional Stud*" OR "Case-Control Stud*" OR "Cohort Stud*" OR "Longitudinal Stud*" OR "Quasi-Experimental Studies" OR "Non-Randomized Controlled Trials" OR "Randomized Controlled Trials" OR "determinant*" OR "determinant factors" OR "Associated factor*" OR "factor*" OR "association" OR "risk factor*" OR "risk" OR "predictor*" OR "driver*")<br>*Additional limits - Source type: Scholarly Journals*
S4	Under-five children
	abstract("Child*" OR "Minor*" OR "under-five child*" OR "under-5 child*" OR "children under 5" OR "Children aged 6-59 months" OR "Preschool" OR "Preschool Child*" OR "Early Childhood" OR "Infants" OR "infant and child" OR "Infants and young children" OR "toddler*" OR "Pre-school age children" OR "early years" OR "young children")
S5	[S1] AND [S2]
S6	[S5] AND [S3]
S7	[S6] AND [S4]

Global Index Medicus SEARCH STRATEGY

("Nutritional Failure" OR "Nutritional Disorder$" OR "Nutritional Deficienc$" OR "Malnourishment$" OR "Protein-Energy Malnutrition" OR "malnourish$" OR "Severe Acute Malnutrition" OR "Undernutrition" OR "Malnourished Children" OR "stunting$" OR "wasting" OR "underweight" OR "chronic undernutrition" OR "wasting and underweight" OR "underweight and stunting" OR "Anthropometric Indices" OR "anthropometric index" OR "anthropometric failure" OR "anthropometric deficits" OR "CIAF" OR "CISAF" OR "ECIAF") AND ("composite index" OR "multiple failures" OR "overall burden" OR "aggregate failure$" OR "coexist$" OR "co-occurring" OR "concurrent$" OR "concomitant") AND ("determinants" OR "determinant$" OR "determinant factors" OR "Associated factor$" OR "factor$" OR "association" OR "risk factor$" OR "risk" OR "predictor$" OR "driver$" OR "factors associated" OR "Prevalence" OR "Prevalences" OR "burden$" OR "Observational Stud$" OR "Cross Sectional Stud$" OR "Case-Control Stud$" OR "Cohort Stud$" OR "Longitudinal Stud$" OR "Quasi-Experimental Studies" OR "Non-Randomized Controlled Trials" OR "Randomized Controlled Trials" AND ("Child$" OR "Minor$" OR "under-five child$" OR "under-5 child$" OR "children under 5" OR "Children aged 6-59 months" OR "Preschool" OR "Preschool Child$" OR "Early Childhood" OR "Pre-school age children")

Cochrane Library SEARCH STRATEGY

	Anthropometric failure
#1	MeSH descriptor: [Child Nutrition Disorders] explode all trees
#2	MeSH descriptor: [Malnutrition] explode all trees
#3	MeSH descriptor: [Severe Acute Malnutrition] explode all trees
#4	MeSH descriptor: [Anthropometry] explode all trees
#5	((Nutritional NEXT (Failure* OR disorder* OR deficienc* OR status)) OR (Nutrition NEXT (Disorder* OR assessment)) OR Malnourishment* OR malnourish* OR (Protein-Energy NEXT Malnutrition) OR (Child NEXT Malnutrition) OR (Malnourished NEXT Children) OR stunting* OR wasting OR underweight OR Undernutrition OR (chronic NEXT undernutrition) OR anthropometric* OR (Anthropometric NEXT (Z-score* OR indices OR index OR failure* OR deficits)) OR (Acute NEXT Malnutrition) OR CIAF OR CISA OR ECIAF):ti,ab,kw
#6	#1 OR #2 OR #3 OR #4 OR #5
	Composite index
#7	(composite index OR multiple failures OR overall NEXT burden OR aggregate NEXT failure* OR coexist* OR co-occurring OR concurrent* OR concomitant OR Syndemic*):ti,ab,kw
#8	MeSH descriptor: [Syndemic] explode all trees
#9	#7 OR #8
	Determinants
#10	MeSH descriptor: [Risk] explode all trees

#11	MeSH descriptor: [Risk Factors] explode all tree
#12	MeSH descriptor: [Epidemiologic Factors] explode all trees
#13	(determinant* OR (determinant NEXT factor*) OR (Associated NEXT factor*) OR factor* OR association OR (risk NEXT factor*) OR risk OR predictor* OR driver*) OR (epidemiology OR Prevalence OR Prevalences OR burden* OR (Prevalence NEXT Stud*) OR (Observational NEXT Stud*) OR (Cross-Sectional NEXT Stud*) OR (Case-Control NEXT Stud*) OR (Cohort NEXT Stud*) OR (Longitudinal NEXT Stud*) OR (Quasi-Experimental NEXT Stud*) OR (Non-Randomized NEXT Trials) OR (Randomized NEXT Trials) :ti,ab,kw
#14	#10 OR #11 OR #12 OR #13
	Under-five children
#15	MeSH descriptor: [Child, Preschool] explode all trees
#16	MeSH descriptor: [Infant] explode all trees
#17	(Child* OR Minor* OR (under-five NEXT child*) OR (under-5 NEXT child*) OR Preschool OR (Preschool NEXT Child*) OR (Early NEXT Childhood) OR Infant* OR (young NEXT children) OR toddler* OR (Pre-school NEXT child*)):ti,ab,kw
#18	#15 OR #16 OR #17
#19	#6 AND #9
#20	#19 AND #14
#21	#20 AND #18
