# Supplementary material for: Socio-ecological determinants of multiple anthropometric failures among under-five children: A systematic review and meta-analysis of observational studies
Source: PLOS Glob Public Health. 2025 Jul 31;5(7):e0005008. doi: 10.1371/journal.pgph.0005008 (PMC12312983; doi:10.1371/journal.pgph.0005008)
Supplement: S1 Table — (PDF) [file pgph.0005008.s003.pdf]

S1_Table: List of articles excluded after full-text review, including the specific reasons for their exclusion.

Sr.no	Title	DOI/URL	Reason
1	Overall burden of under-nutrition measured by a Composite Index in rural pre-school children in Purba Medinipur, West Bengal, India	http://dx.doi.org/10.2478/anre-2013-0005	Not a study population/ Mixed age group (e.g.: 3 to 6 years/ 0-6yr/
2	Composite Index of Anthropometric Failure and its correlates: a cross-sectional study of under five children in an urban informal settlement of Mumbai	https://www.snehamumbai.org/wp-content/uploads/2021/04/Composite-Index-of-Anthropometric-Failure-Report.pdf	Not Peer Reviewed
3	Re-estimating malnourishment and inequality among children in north-east India	https://www.jstor.org/stable/24479264	Similar dataset was used
4	The concept of composite index of anthropometric failure (CIAF): revisited and revised. Anthropol-Open J. 2018; 4 (1): 3 (1)–35	http://dx.doi.org/10.17140/ANTPOJ-3-118	Review
5	Assessment of nutritional status by composite index for anthropometric failure: a study among slum children in Bankura, West Bengal	http://dx.doi.org/10.4103/0019-557x.106421	Short communication
6	Nutritional status among boys and girls of a central Indian Town (Sagar)	http://dx.doi.org/10.1515/anre-2015-0014	Age >5yr (Not the study population)
7	Household food insecurity and nutritional status of schoolchildren in rural regions of Bajo Lampa, El Salvador (2018-2019)	http://dx.doi.org/10.1080/03670244.2021.1968851	Age >5yr (Not the study population)
8	Extent of Nutritional failure among under-five children from an urban slum in South India: A cross-sectional assessment using Composite index of Anthropometric failure	https://doi.org/10.21203/rs.3.rs-2433822/v1	Not Peer Reviewed
9	Assessment of undernutrition using composite index of anthropometric failure among children less than 5 years in an urban slum, Visakhapatnam	http://dx.doi.org/10.18203/2394-6040.ijcmph20184567	Methodological problems (small sample size)
10	Determinants of coexisting forms of undernutrition among under-five children: Evidence from the Bangladesh demographic and health surveys	http://dx.doi.org/10.1002/fsn3.3484	Not relevant to the study
11	Nutritional status of Mid-Day Meal programme beneficiaries: A cross-sectional study among primary schoolchildren in Kottayam district, Kerala, India	http://dx.doi.org/10.4103/ijph.IJPH_320_15	Age >5yr (Not the study population)
12	Assessment of Nutritional Status of Under-Five Children in an Urban Area of South Delhi, India	http://dx.doi.org/10.7759/cureus.34924	Health facility-based study/ Methodological problems
13	Socio-Economic And Demographic Correlates Of Composite Index Of Anthropometric Failure Among Rural Children In West Bengal, India	https://www.arfjournals.com/image/catalog/Journals%20Papers/MII/2020/No.1-2/05.pdf	Age >5yr (Not the study population)
14	Malnutrition in Infants Aged under 6 Months Attending Community Health Centres: A Cross Sectional Survey	http://dx.doi.org/10.3390/nu13082489	Health facility-based study/ Methodological problems
15	Mother's education level is associated with anthropometric failure among 3- to 12-year-old rural children in Purba Medinipur, West Bengal, India	http://dx.doi.org/10.1017/s0021932020000577	Not a study population /Mixed age group (e.g.: 3 to 6 years/ 0-6yr/)
16	Anthropometric failures and its predictors among under five children in Ethiopia: multilevel logistic regression model using 2019 Ethiopian demographic and health survey data	http://dx.doi.org/10.1186/s12889-024-18625-4	Similar dataset was used
17	Concurrently wasted and stunted children 6-59 months in Karamoja, Uganda: prevalence and case detection	http://dx.doi.org/10.1111/mcn.13000	Not relevant to the study objective
18	Double burden of malnutrition among children under 5 in poor areas of China	http://dx.doi.org/10.1371/journal.pone.0204142	Not relevant to the study objective
19	Assessment of under nutrition using composite index of anthropometric failure among under five children of tribal population	http://dx.doi.org/10.18203/2394-6040.ijcmph20191818	Methodological problems
20	Composite Index of Anthropometric Failure (CIAF) among Juang children and adolescents of Keonjhar district in Odisha, India	http://dx.doi.org/10.47509/MES.2022.v03i01.10	Age >5yr (Not the study population)

**21**	Are we Underestimating the Real Burden of Malnutrition? An Experience From Community-Based Study	http://dx.doi.org/10.4103/0970-0218.164401	Short Communication
**22**	Assessment of malnutrition using Z-scores and Composite Index of Anthropometric Failure among street children in Delhi	http://dx.doi.org/10.1016/j.nut.2024.112487	Age >5yr (Not the study population)
**23**	An intersectional analysis of the composite index of anthropometric failures in India	http://dx.doi.org/10.1186/s12939-021-01499-y	Similar dataset was used
**24**	Assessment of undernutrition by composite index of anthropometric failure among under five children in a slum of Kolkata, West Bengal	http://dx.doi.org/10.18203/2394-6040.ijcmph20201449	Methodological problems
**25**	A community-based cross-sectional study to assess the burden of prevalence of undernutrition in 0-6 Years Anganwadi Children of Panvel Block, Raigad District, Maharashtra	http://dx.doi.org/10.1007/s12098-023-04865-6	Not relevant to the study objective
**26**	Minimum Acceptable Diet, Anthropometric Failure and Correlates among Children Aged 6-23 Months in a Rural Area of Murshidabad, West Bengal	http://dx.doi.org/10.55489/njcm.150620243832	Methodological problems
**27**	Prevalence of under-nutrition measured by Composite Index of Anthropometric Failure (CIAF) among the Bhumij children of Northern Odisha, India	http://dx.doi.org/10.3126/jnps.v36i1.14390	Not a study population /Mixed age group (Age 1 to 6)
**28**	Incidence of Asymptomatic Shigella Infection and Association with the Composite Index of Anthropometric Failure among Children Aged 1–24 Months in Low-Resource Settings	https://doi.org/10.3390/life12050607	Not relevant to the study objective
**29**	Determinants of under-nutrition among children under five years of age in Ethiopia	http://dx.doi.org/10.1186/s12889-020-08539-2	Similar dataset was used
**30**	Child undernutrition in the states of india: an analysis based on change in composite index of anthropometric failure from 2006 to 2016	http://dx.doi.org/10.1177/09722661211010376	Similar dataset was used
**31**	Concurrent wasting and stunting among under-five children in Niakhar, Senegal	http://dx.doi.org/10.1111/mcn.12736	Not relevant to the study objective
**32**	A Comparison: Composite Index of Anthropometric Failure (CIAF) Incidence in Bukittinggi City and Dharmasraya District, Indonesia	http://dx.doi.org/10.4108/eai.9-10-2019.2297252	Not relevant to the study objective
**33**	The Composite Index of Anthropometric Failure (CIAF): An Alternative Indicator for Malnutrition in Young Children	http://dx.doi.org/10.1007/978-1-4419-1788-1_6	Book chapter
**34**	Assessing Burden of Under-Nutrition among Underfive Children of Urban Slum by Using Composite Index of Anthropometric Failure in Ahmedabad City, Gujarat, India		Not relevant to the study objective
**35**	Levels and trends of childhood undernutrition by wealth and education according to a composite index of anthropometric failure: evidence from 146 demographic and health surveys from 39 countries	http://dx.doi.org/10.1136/ bmjgh-2016-000206	Not relevant to the study objective
**36**	Composite index of anthropometric failure and its important correlates: a study among under-5 children in a slum of Kolkata, West Bengal, India	http://dx.doi.org/10.5455/ijmsph.2015.0111201485	Not relevant to the study objective
**37**	Food security and anthropometric failure among tribal children in Bankura, West Bengal	http://dx.doi.org/10.1007/s13312-011-0057-2	Short Communication
**38**	Stunting among primary-school children: a sample from Baghdad, Iraq	https://applications.emro.who.int/emhj/1502/15_2_2009_0322_0329.pdf	Not a study population/ Mixed age group (e.g.: 3 to 6 years/ 0-6yr/)
**39**	Composite Index of Anthropometric Failure (CIAF): A Better Indicator of Overall Burden of Undernutrition Among Primary School Children	http://dx.doi.org/10.4038/sljch.v50i2.9553	Age >5yr (Not the study population)
**40**	Malnutrition in primary school-age children: A case of urban and slum areas of Bahawalpur, Pakistan	http://dx.doi.org/10.1108/03068291111157221	Age >5yr (Not the study population)

**41**	Composite Index of Anthropometric Failure (CIAF) among preschool (2–5 years) tribal children of Assam (India)	https://www.researchgate.net/publication/322910313_Composite_Index_of_Anthropometric_Failure_CIAF_among_pre-school_2-5_years_tribal_children_of_Assam_India	Not relevant to the study
**42**	Mortality risk in infants receiving therapeutic care for malnutrition: A secondary analysis	http://dx.doi.org/10.1111/mcn.13635	Health facility based study/ Methodological problems
**43**	Proposed new anthropometric indices of childhood undernutrition	https://maljnutr.org.my/publication/16-1/k.pdf	Not a study population/ Mixed age group (e.g.: 3 to 6 years/ 0-6yr/)
**44**	Assessment of undernutrition using the composite index of anthropometric failure (CIAF) and its determinants: A cross-sectional study in the rural area of the Bogor District in Indonesia	http://dx.doi.org/10.1186/s40795-022-00627-3	Duplicated Article
**45**	Assessment Of Undernutrition Using Composite Index Of Anthropometric Failure (Ciaf) And Conventional Anthropometric Indices Among Anganwadi Children (2-5 Years) Of Raipur City, Chhattisgarh, India	https://utkaluniversity.ac.in/wp-content/uploads/2023/02/Man-in-Society-Department-of-Anthropology-UU.pdf#page=29	Not Peer Reviewed
**46**	Conventional nutritional indices and Composite Index of anthropometric failure: Which seems more appropriate for assessing under-nutrition among children? A cross-sectional study among school children of the Bengalee Muslim population of North Bengal, India	https://doi.org/10.2427/5659	Age >5yr (Not the study population)
**47**	Overlooking undernutrition? Using a composite index of anthropometric failure to assess how underweight misses and misleads the assessment of undernutrition in young children	http://dx.doi.org/10.1016/j.socscimed.2008.01.021	Short Communication
**48**	Comparison of WHO 2006 Growth Standards and Synthetic Indian References in Assessing Growth in Normal Children and Children with Growth-Related Disorders	http://dx.doi.org/10.4103/ijem.ijem_380_23	Not relevant to the study objective
**49**	Space-time dynamics regression models to assess variations of composite index for anthropometric failure across the administrative zones in Ethiopia	http://dx.doi.org/10.1186/s12889-022-13939-7	Similar dataset was used
**50**	Prevalence and correlates of the composite index of anthropometric failure among children under 5 years old in Bangladesh	http://dx.doi.org/10.1111/mcn.12930	Similar dataset was used
**51**	Use of modified composite index of anthropometric failure and MUAC-for-age to assess prevalence of malnutrition among school-age children and adolescents involved in the school feeding program in Addis Ababa, Ethiopia	http://dx.doi.org/10.1186/s40795-021-00471-x	Age >5yr (Not the study population)
**52**	Composite index of anthropometric failure among anganwadi children in rural field practice area of Vydehi Institute of Medical Sciences and Research Centre	http://dx.doi.org/10.9790/0853-1503070913	Methodological problems
**53**	Z-score and CIAF–A descriptive measure to determine prevalence of under-nutrition in rural school children, Puducherry, India	http://dx.doi.org/10.7860/JCDR/2018/22224.11560	Age >5yr (Not the study population)
**54**	Prevalence and determinants of concurrent wasting and stunting and other indicators of malnutrition among children 6–59 months old in Kersa, Ethiopia	http://dx.doi.org/10.1111/mcn.13172	Not relevant to the study objective
**55**	Socio-economic and demographic factors affecting the Composite Index of Anthropometric Failure (CIAF)	http://dx.doi.org/10.3109/03014460.2012.655777	Not a study population /Mixed age group (e.g.: 3 to 6 years/ 0-6yr/)
**56**	Extent and spread of malnutrition among children under five in Indian states	http://dx.doi.org/10.1007/s10708-024-11050-5	Not relevant to the study
**57**	Changes in Child Nutrition in India: A Decomposition Approach	http://dx.doi.org/10.3390/ijerph16101815	Not relevant to the study
**58**	Optimistic factors affecting nutritional status among children during early childhood in rural areas of western China	https://pubmed.ncbi.nlm.nih.gov/17767873/	Not relevant to the study
**59**	Undernutrition Among the Children from Different Social Groups in India: Prevalence, Determinants, and Transition Over Time (2005-2006 to 2019-2021)	https://doi.org/10.1007/s40615-023-01796-y	Prevalence data only/ Not relevant to the study objective

**60**	Socio-Economic Determinants of Child Under-Nutrition in Pakistan: A Measurement of Composite Index of Anthropometric Failure Using PDHS Micro Data	https://doi.org/10.47205/jdss.2022(3-III)80	Prevalence data only/ Not relevant to the study objective
**61**	Disparities in childhood composite index of anthropometric failure prevalence and determinants across Ethiopian administrative zones	http://dx.doi.org/10.1371/journal.pone.0256726	Prevalence data only/ Not relevant to the study objective
**62**	Examining the changing profile of undernutrition in the context of food price rises and greater inequality	http://dx.doi.org/10.1016/j.socscimed.2015.11.036	Prevalence data only/ Not relevant to the study objective
**63**	Analysis of Malnutrition among Children under Five Years across Contrasting Agroecosystems of Northwest Ethiopia: Application of Structural Equation Modeling	http://dx.doi.org/10.3390/nu16081208	Prevalence data only/ Not relevant to the study objective
**64**	Prevalence of Undernutrition and Anemia among Santal Adivasi Children, Birbhum District, West Bengal, India	http://dx.doi.org/10.3390/ijerph17010342	Prevalence data only/ Not relevant to the study objective
**65**	Assessment of Under Nutrition Using Composite Index of Anthropometric Failure (CIAF) amongst Toddlers Residing in Urban Slums of Raipur City, Chhattisgarh, India	http://dx.doi.org/10.7860/JCDR/2015/12822.6197	Prevalence data only/ Not relevant to the study objective
**66**	Estimated burden of aggregate anthropometric failure among Malawian children	https://doi.org/10.1080/16070658.2017.1387433	Prevalence data only/ Not relevant to the study objective
**67**	Deconstructing the sex gap in child undernutrition in India: Are Indian boys at elevated risk of anthropometric failure than the girls?	http://dx.doi.org/10.1002/ajhb.24092	Prevalence data only/ Not relevant to the study objective
**68**	The Effect of “Women’s Empowerment” on Child Nutritional Status in Pakistan	http://dx.doi.org/10.3390/ijerph16224499	Prevalence data only/ Not relevant to the study objective
**69**	Extended composite index of anthropometric failure in Argentinean preschool and school children	http://dx.doi.org/10.1017/S1368980019002027	Not relevant to the study objective, focus on extended CIAF
**70**	Prevalence and correlates of severe under-5 child anthropometric failure measured by the composite index of severe anthropometric failure in Bangladesh	http://dx.doi.org/10.3389/fped.2022.978568	Not relevant to the study objective, focus on severe failure
**71**	Unraveling the South Asian enigma: concurrent manifestations of child anthropometric failures and their determinants in selected South Asian countries	http://dx.doi.org/10.1186/s40795-023-00771-4	Not relevant to the study objective
**72**	Composite Index of Anthropometric Failure and Early Childhood Cognitive Development Based on the 2018 Indonesian Basic Health Research Data	https://scholarhub.ui.ac.id/kesmas/vol18/iss4/4	Not relevant to the study objective
**73**	Determinants Of Malnutrition Based On The Composite Index Of Anthropometric Failure (CIAF)	https://jurnalbidankestrad.com/index.php/jkk/article/view/389	Prevalence data only/ Not relevant to the study objective
**74**	Measuring undernutrition through z-scores and Composite Index of Anthropometric Failure (CIAF): a study among slum children in Ahmedabad City, Gujarat	https://njcmindia.com/index.php/file/index	Prevalence data only/ Not relevant to the study objective
**75**	Magnitude of undernutrition in children aged 2 to 4 years using CIAF and conventional indices in the slums of Mumbai city	https://doi.org/10.1186/s41043-015-0017-x	Prevalence data only
**76**	Composite Index of Anthropometric Failure (CIAF) among Sonowal Kachari tribal preschool children of flood effected region of Assam, India	https://doi.org/10.2478/anre-2019-0012	Prevalence data only/ Not relevant to the study objective
**77**	Determinants of malnutrition in Indian children: new evidence from IDHS through CIAF	https://doi.org/10.1007/s11135-014-0149-x	Prevalence data only/ Not relevant to the study objective
**78**	Composite Index of Anthropometric Failure among below five Children of Korba Block, Chhattisgarh, India	https://antrocom.net/wp/wp-content/uploads/2024/05/dhansay-anthropometric-failure-children-korba-block-chhattisgarh.pdf	Prevalence data only/ Not relevant to the study objective
**79**	Measuring Malnutrition -The Role of Z Scores and the Composite Index of Anthropometric Failure (CIAF)	http://dx.doi.org/10.4103/0970-0218.53392	Prevalence data only/ Not relevant to the study objective

**80**	Nutritional Status of Children in Bangladesh: Measuring Composite Index of Anthropometric Failure (CIAF) and its Determinants	https://mpra.ub.uni-muenchen.de/66550/1/MPRA_paper_66550.pdf	Prevalence data only/ Not relevant to the study objective
**81**	Prevalence of Malnutrition in Human Immunodeficiency Virus/Acquired Immunodeficiency Syndrome Orphans in the Nyanza Province of Kenya: A Comparison of Conventional Indexes with a Composite Index of Anthropometric Failure	http://dx.doi.org/10.1016/j.jada.2008.03.008	Prevalence data only/ Not relevant to the study objective
**82**	Analysis of the Relationship Between Children's Characteristics, Family Characteristics, Food Intake, Eating Habits, and Disease History with Nutritional Status of Under-Five Children Based on the Composite Index of Anthropometric Failure in Karangkamulyan Village, Cihara District, Lebak Regency, Indonesia in 2020	https://scholarhub.ui.ac.id/ijphn/vol3/iss1/1/	Prevalence data only/ Not relevant to the study objective
**83**	Burden of Undernutrition, Composite Index of Anthropometric Failure (CIAF) and Perception of Caregivers about Undernutrition Among Under Five Children in Rural India	https://informaticsjournals.co.in/index.php/ijnd/article/view/2456	Prevalence data only/ Not relevant to the study objective
**84**	Magnitude and severity of anthropometric failure among children under two years using Composite Index of Anthropometric Failure (CIAF) and WHO standards	https://doi.org/10.1016/j.ijpam.2017.12.003	Prevalence data only/ Not relevant to the study objective
**85**	An intersectional analysis of the composite index of anthropometric failures in India	https://doi.org/10.1186/s12939-021-01499-y	Prevalence data only/ Not relevant to the study objective
**86**	Assessment of undernutrition using Composite Index of Anthropometric Failure among children aged < 5 years in rural Yemen	https://doi.org/10.26719/2018.24.12.1119	Prevalence data only/ Not relevant to the study objective
**87**	Individual and Ecological Variation in Child Undernutrition in India: A Multilevel Analysis	https://doi.org/10.1177/0973174115588841	Prevalence data only/ Not relevant to the study objective
**88**	Comparison of Different Nutritional Screening Approaches and the Determinants of Malnutrition in Under-Five Children in a Marginalized District of Punjab Province, Pakistan	https://doi.org/10.3390/children9071096	Prevalence data only/ Not relevant to the study objective
**89**	Assessing Progress towards SDG2: Trends and Patterns of Multiple Malnutrition in Young Children under 5 in West and Central Africa	https://doi.org/10.1007/s12187-019-09671-1	Prevalence data only/ Not relevant to the study objective
**90**	Identification of undernutrition in under five children: Z score or a composite index of anthropometric failure	https://doi.org/10.18203/2394-6040.ijcmph20192866	Prevalence data only/ Not relevant to the study objective
**91**	The burden of anthropometric failure and child mortality in India	https://doi.org/10.1038/s41598-020-76884-8	Prevalence data only/ Not relevant to the study objective
**92**	Changes in Child Nutrition in India: A Decomposition Approach	https://doi.org/10.3390/ijerph16101815	Prevalence data only/ Not relevant to the study objective
**93**	Impact of integrated preventive and curative health package on nutritional status of children under 2 years of age in the health area of Tama, Tahoua region (Niger)	https://doi.org/10.3389/fnut.2023.1259706	Prevalence data only/ Not relevant to the study objective
**94**	The interrelationships of child under-nutrition, ecological and maternal factors: A case study of Pakistan by using composite index of anthropometric failure	https://www.aloki.hu/pdf/1706_1303513055.pdf	Prevalence data only/ Not relevant to the study objective
**95**	Comparison of Child Undernutrition Anthropometric Indicators Across 56 Low- and Middle-Income Countries	https://doi.org/10.1001/jamanetworkopen.2022.1223	Prevalence data only/ Not relevant to the study objective
**96**	Composite Index of Anthropometric Failure (CIAF) among preschool (2-5 years) tribal children of Assam (India)	https://www.humanbiologyjournal.com/article/composite-index-of-anthropometric-failure-ciaf-among-pre-school-2-5-years-tribal-children-of-assam-india/	Prevalence data only/ Not relevant to the study objective

**97**	Composite index of anthropometric failure and geographic altitude in children from Jujuy (1 to 5 years old)	https://doi.org/10.5546/aap.2014.eng.526	Prevalence data only/ Not relevant to the study objective
**98**	Acute childhood morbidities in rural Wardha: some epidemiological correlates and health care seeking	https://www.bioline.org.br/pdf?ms09062	Prevalence data only/ Not relevant to the study objective
**99**	Assessment of nutritional status by composite index of anthropometric failure (CIAF): a study among preschool children of Sagar Block, South 24 Parganas District, West Bengal, India	https://doi.org/10.2478/anre-2018-0022	Prevalence data only/ Not relevant to the study objective
**100**	Report on "anthropometric failure" among rural 2-6 years old Indian Bauri caste children of West Bengal	https://doi.org/10.2478/v10044-008-0017-1	Not a study population/ Mixed age group/
**101**	Prevalence and factors associated with severe undernutrition among under-5 children in Bangladesh, Pakistan, and Nepal: a comparative study using multilevel analysis	https://doi.org/10.1038/s41598-023-36048-w	Prevalence data only/ Not relevant to the study objective
**102**	Early childhood undernutrition, preadolescent physical growth, and cognitive achievement in India: A population-based cohort study	https://doi.org/10.1371/journal.pmed.1003838	Prevalence data only/ Not relevant to the study objective
**103**	Urban-rural differences in the associated factors of severe under-5 child undernutrition based on the composite index of severe anthropometric failure (CISAF) in Bangladesh	https://doi.org/10.1186/s12889-021-12038-3	Prevalence data only/ Not relevant to the study objective
**104**	Nutritional Status of One to Five Year Old Children in Rural Haryana: A Community Based Study	https://doi.org/10.7860/JCDR/2020/45635.14045	Prevalence data only/ Not relevant to the study objective
**105**	Measuring the Overall Burden of Early Childhood Malnutrition in Ghana: A Comparison of Estimates From Multiple Data Sources	https://doi.org/10.34172/ijhpm.2020.253	Prevalence data only/ Not relevant to the study objective
**106**	Assessment of Overall Prevalence of Undernutrition Using Composite Index of Anthropometric Failure (CIAF) among Preschool Children of West Bengal, India	https://www.bioline.org.br/pdf?pe09036	Prevalence data only/ Not relevant to the study objective
**107**	Overall Prevalence of Undernutrition Measured by Composite Index of Anthropometric Failure (CIAF): A Study among the Rural and Urban Preschool Children of West Bengal, India	https://www.sid.ir/paper/555920/en	Prevalence data only/ Not relevant to the study objective
**108**	Composite Index of Anthropometric Failure among 2-5 years Anganwadi Children of Bilaspur, Chhattisgarh, India	https://antrocom.net/wp/wp-content/uploads/2024/05/ghritlahre-das-anthropometric-failure-anganwadi-children-chhattisgarh.pdf	Prevalence data only/ Not relevant to the study objective
**109**	Assessment of Nutritional Status of Underfive Children in a Low Socio-Economic Urban Community of Guntur city in AP State	https://doi.org/10.37506/ijphrd.v11i11.11342	Prevalence data only/ Not relevant to the study objective
**110**	Socio-demographic risk factors for severe malnutrition in children aged under five among various birth cohorts in Bangladesh	https://doi.org/10.1017/S0021932020000425	Prevalence data only/ Not relevant to the study objective
**111**	Assessment of Nutritional Status by Anthropometric Indices in Santal Tribal Children	https://doi.org/10.1080/09751270.2011.11885172	Prevalence data only/ Not relevant to the study objective
**112**	Measuring undernutrition by composite index of anthropometric failure (CIAF): a community-based study in a slum of Nagpur city	https://www.bibliomed.org/?mno=218138	Prevalence data only/ Not relevant to the study objective
**113**	Can we rely solely on conventional measures to estimate undernutrition among under-fives?	https://www.iapsmupuk.org/journal/index.php/IJCH/article/view/585	Prevalence data only/ Not relevant to the study objective
**114**	Socio-demographic Risk Factors of Child Undernutrition	https://doi.org/10.17334/jps.96514	Prevalence data only/ Not relevant to the study objective
**115**	Prevalence of undernutrition among pre-school children of Chapra, Nadia District, West Bengal, India, measured by composite index of anthropometric failure (CIAF)	https://doi.org/10.1127/0003-5548/2009/0025	Prevalence data only/ Not relevant to the study objective

**116**	Nutritional assessment among children (1–5 years of age) using various anthropometric indices in a rural area of Haryana, India	https://doi.org/10.4103/IJCFM.IJCFM_14_19	Prevalence data only/ Not relevant to the study objective
**117**	Regional Differences of Child Undernutrition in India: The Reflection of Composite Index of Anthropometric Failure (CIAF) from CNNS-2016-18 Data	https://imsear.searo.who.int/bitstreams/07dd2644-007c-4c20-90ff-889b1e122fb9/download	Prevalence data only/ Not relevant to the study objective
**118**	Anthropometric failure, a new approach to measure undernutrition: An experience from a rural community of West Bengal, India	https://www.researchgate.net/publication/26877278_Anthropometric_failure_a_new_approach_to_measure_undernutrition_An_experience_from_a_rural_community_of_West_Bengal_India	Prevalence data only/ Not relevant to the study objective
**119**	Are Household Food Security, Nutrient Adequacy, and Childhood Nutrition Clustered together? A Cross-Sectional Study in Bankura, West Bengal	https://doi.org/10.4103/ijph.IJPH_357_18	Prevalence data only/ Not relevant to the study objective
**120**	Composite Index of Anthropometric Failure among preschool children under ICDS of Jalpaiguri District, West Bengal, India	https://doi.org/10.62424/IJBS.2022.28.01.03	Prevalence data only/ Not relevant to the study objective
**121**	Association of trends in child undernutrition and implementation of the National Rural Health Mission in India: A nationally representative serial cross-sectional study on data from 1992 to 2015	https://doi.org/10.1371/journal.pmed.1003957	Prevalence data only/ Not relevant to the study objective
**122**	Public Health Implications of Wasting and Stunting Relationship in Children under Five Years Highly Vulnerable to Undernutrition in Guatemala	https://doi.org/10.3390/nu14193945	Prevalence data only/ Not relevant to the study objective
**123**	Developing and demonstrating an atomistic and holistic model of anthropometric failure among children under five years of age using the National Family Health Survey (NFHS)-5 data from India.	https://doi.org/10.3389/fnut.2023.1280219	Prevalence data only/ Not relevant to the study objective
**124**	Composite indexes of anthropometric failure in children under 5 years of age in Argentina: Comparative analysis among regions: 2019–2020	https://doi.org/10.1002/ajhb.23994	Prevalence data only/ Not relevant to the study objective
**125**	Comparison of Nutritional Status of Healthy Under-Five Indian Children Using Composite Index of Anthropometric Failure on WHO 2006 versus 2019 Indian Synthetic Growth Charts	https://doi.org/10.1007/s12098-023-04865-6	Prevalence data only/ Not relevant to the study objective
**126**	Assessment by neo-CIAF formula predicts contrast occurrence of overweight and undernourishment in preschool children of Jangalmahal districts, India	https://doi.org/10.59213/TP.2024.183	Prevalence data only/ Not relevant to the study objective
**127**	Assessment of Undernutrition Among Under-Five Children by Using Composite Index of Anthropometric Failure (CIAF)	https://doi.org/10.70135/seejph.vi.2498	Prevalence data only/ Not relevant to the study objective
