# Supplementary material for: Socio-ecological determinants of multiple anthropometric failures among under-five children: A systematic review and meta-analysis of observational studies
Source: PLOS Glob Public Health. 2025 Jul 31;5(7):e0005008. doi: 10.1371/journal.pgph.0005008 (PMC12312983; doi:10.1371/journal.pgph.0005008)
Supplement: S2 Table — (PDF) [file pgph.0005008.s004.pdf]

S2_Table: Quality Assessment of Included Studies Using the Adapted Newcastle-Ottawa Scale for Cross-Sectional Studies[1]

Authors name, Year of publication	Selection (Maximum 5 stars)				Comparability (Maximum 2 stars)	Outcome (Maximum 3 stars)		Total	Risk of bias
	Representativeness of the sample	Sample size	Non-respondents	Ascertainment of the exposure (risk factor)	Confounding factors are controlled	Assessment of the outcome	Statistical test		
Shah G et al, 2024	*	*	*	*	**	**	*	9	Very Good
Kundu RN et al, 2023	*	*	*	*	**	**	*	9	Very Good
Soni A et al, 2022	*	*	*	*	**	**	*	9	Very Good
Khamis AG et al, 2020	*	*	*	*	**	**	*	9	Very Good
Ghosh P, 2023	*	*	*	*	**	**	*	9	Very Good
Porwal A et al, 2021	*	*	*	*	**	**	*	9	Very Good
Chowdhury MRK et al, 2021	*	*	*	*	**	**	*	9	Very Good
Alarape K et al, 2022	*	*	*	*	**	**	*	9	Very Good
Naveed TA et al, 2022	*	*	*	*	**	**	*	9	Very Good
Kundu RN et al, 2022	*	*	*	*	**	**	*	9	Very Good
Pei L et al, 2014	*	*	*	*	**	**	*	9	Very Good
Amusal LB et al, 2023	*	*	*	*	**	**	*	9	Very Good
Sahiledengle B et al, 2024	*	*	*	*	**	**	*	9	Very Good
Ali Khan RE et al, 2014	*	*	*	*	**	**	*	9	Very Good
Siddiqa M et al, 2024	*	*	*	*	**	**	*	9	Very Good
Endris N et al, 2017	*	*	*	*	**	**	*	9	Very Good
Balogun OS et al, 2021	*	*	*	*	**	**	*	9	Very Good
Das R et al, 2022	*	*	*	*	**	**	*	9	Very Good
Latifah HI et al, 2024	*	*	*	*	**	**	*	9	Very Good
Shahid M et al, 2022 (b)	*	*	*	*	**	**	*	9	Very Good
Sapkota S et al, 2018	*	*	*	*	**	**	*	9	Very Good
Rathi SK et al, 2024	*	*	*	*	**	**	*	9	Very Good
Jeyakumar A et al, 2021	*	*	*	*	**	**	*	9	Very Good
Bidiral K et al, 2021	*	*	*	*	**	**	*	9	Very Good
Kumari T et al, 2024	*	*	*	*	**	**	*	9	Very Good
Talapalliwar MR et al, 2014	*	*	*	*	**	**	*	9	Very Good
Berra WG, 2020	*	*	*	*	**	**	*	9	Very Good
Berra WG et al, 2020	*	*	*	*	**	**	*	9	Very Good
Shahid M et al, 2022 (a)	*	*	*	*	**	**	*	9	Very Good
Permatasari TAE et al, 2023		*	*	*	**	**	*	8	Good
Gebretsadik MT et al, 2023	*	*	*	*	**	**	*	9	Very Good
Sabu KU et al, 2020	*	*	*	*	**	**	*	9	Very Good
Mohandas A et al, 2023	*	*	*	*	**	**	*	9	Very Good
How ETC et al, 2020	*	*	*	*	**	**	*	9	Very Good
Indris A et al, 2021		*	*	*	**	**	*	8	Good
Manjula M et al, 2017	*	*	*	*	**	**	*	9	Very Good
Jana D et al, 2024	*	*	*	*	**	**	*	9	Very Good

Roy K et al, 2018	*	*	*	*	**	**	*	9	Very Good
Workie DL et al, 2021	*	*	*	*	**	**	*	9	Very Good
Seboka BT et al, 2021	*	*	*	*	**	**	*	9	Very Good
Fenta HM et al, 2021	*	*	*	*	**	**	*	9	Very Good
Vanderhout SM et al, 2020	*	*	*	*	**	**	*	9	Very Good
Salazar Burgos et al, 2024	*	*	*	*		**	*	7	Good
Wubetie BY et al, 2024	*	*	*	*		**	*	7	Good
Stiller CK et al, 2020	*	*	*	*		**	*	7	Good
Shafiq A et al, 2019	*	*	*	*		**	*	7	Good
Savanur MS et al, 2015	*	*	*	*		**	*	7	Good
Bharali N et al, 2019	*	*	*	*		**	*	7	Good
Khan and Raza, 2014	*	*	*	*		**	*	7	Good
Anisadiyah A et al, 2022	*	*	*	*		**	*	7	Good
Permatasari TAE et al, 2022	*	*	*	*		**	*	7	Good
Al-Sadeeq AH et al, 2018	*	*	*	*		**	*	7	Good
Asif MA et al, 2019	*	*	*	*		**	*	7	Good
Bejarano IF et al, 2014	*	*	*	*		**	*	7	Good
Sonil A et al, 2021	*	*	*	*		**	*	7	Good
Mandal GC et al, 2009	*	*	*	*		**	*	7	Good

NEWCASTLE - OTTAWA QUALITY ASSESSMENT SCALE (Adapted for Cross Sectional Studies)

Selection: (Maximum 5 stars)

1) Representativeness of the sample:
   - a) Truly representative of the average in the target population. * (all subjects or random sampling)
   - b) Somewhat representative of the average in the target population. * (non-random sampling)
   - c) Selected group of users.
   - d) No description of the sampling strategy.
2) Sample size:
   - a) Justified and satisfactory. *
   - b) Not justified.
3) Non-respondents:
   - a) Comparability between respondents and non-respondents' characteristics is established, and the response rate is satisfactory. *
   - b) The response rate is unsatisfactory, or the comparability between respondents and non-respondents is unsatisfactory.
   - c) No description of the response rate or the characteristics of the responders and the non-responders.
4) Ascertainment of the exposure (risk factor):
   - a) Validated measurement tool. **
   - b) Non-validated measurement tool, but the tool is available or described. *
   - c) No description of the measurement tool.

Comparability: (Maximum 2 stars)

1) The subjects in different outcome groups are comparable, based on the study design or analysis. Confounding factors are controlled.
   - a) The study controls for the most important factor (select one). *
   - b) The study control for any additional factor. *

Outcome: (Maximum 3 stars)

1) Assessment of the outcome:
   a) Independent blind assessment. **
   b) Record linkage. **
   c) Self report. *
   d) No description.
2) Statistical test:
   a) The statistical test used to analyse the data is clearly described and appropriate, and the measurement of the association is presented, including confidence intervals and the probability level (p value). *
   b) The statistical test is not appropriate, not described or incomplete.

[1]*Stang A: Critical evaluation of the Newcastle-Ottawa scale for the assessment of the quality of nonrandomized studies in meta-analyses. European journal of epidemiology 2010, 25(9):603-605.*
