# Supplementary material for: Socio-ecological determinants of multiple anthropometric failures among under-five children: A systematic review and meta-analysis of observational studies
Source: PLOS Glob Public Health. 2025 Jul 31;5(7):e0005008. doi: 10.1371/journal.pgph.0005008 (PMC12312983; doi:10.1371/journal.pgph.0005008)
Supplement: S1 Fig — (PDF) [file pgph.0005008.s005.pdf]

S1_Fig: Forest plots illustrating associations between child, household, and environmental factors and CIAF among children under five

Contents

Fig A: Forest plot of the association between anemia and CIAF among children under five 2

Fig B: Forest plot of the association between moderate/severe forms of anaemia and CIAF among children under five 2

Fig C: Forest plot illustrating the relationship between a history of diarrhea in children and CIAF 3

Fig D: Forest plot illustrating the relationship between a history of fever/ARI in children and CIAF 3

Fig E: Forest plot illustrating the relationship between comorbidity in children and CIAF 3

Fig F: Forest plot illustrating the relationship between milk consumption and CIAF 4

Fig G: Forest plot illustrating the relationship between breastfeeding and CIAF 4

Fig H: Forest plot illustrating the relationship between family size and CIAF 4

Fig I: Forest plot illustrating the association between household food security and CIAF among children under five 5

Fig J: Forest plot illustrating the association between place of residency and CIAF 5

Fig K: Forest plot illustrating the association between sanitation facility and CIAF 5

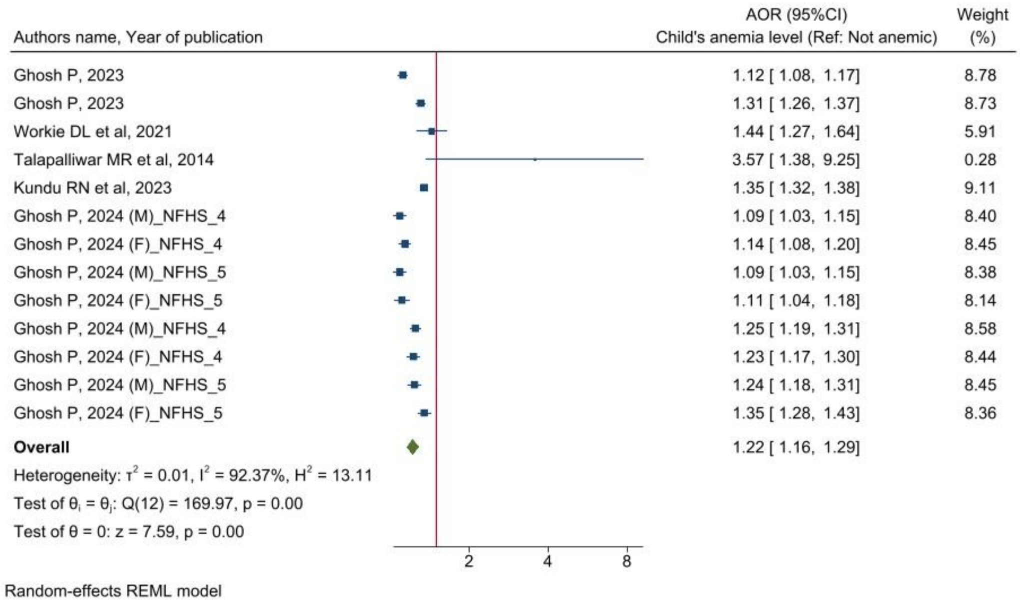

Fig A: Forest plot of the association between anemia and CIAF among children under five

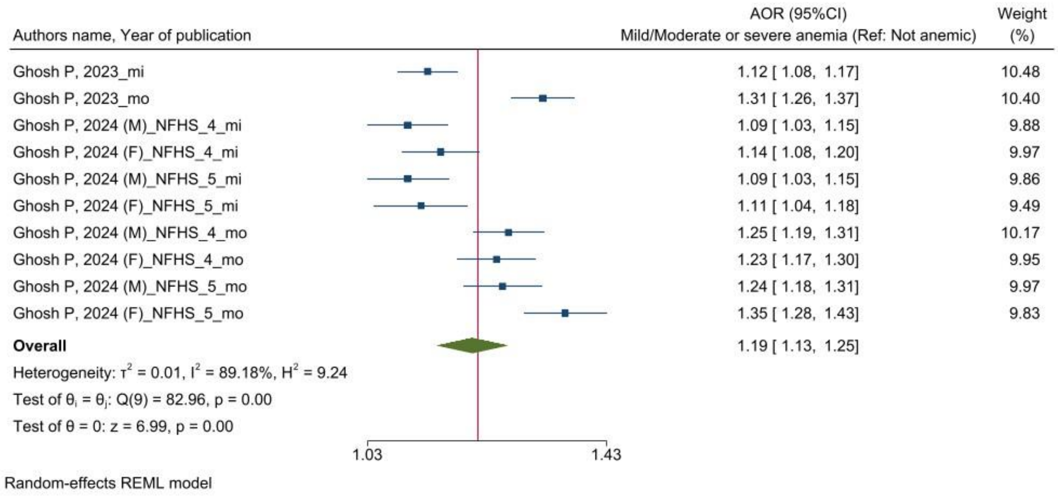

Fig B: Forest plot of the association between moderate/severe forms of anaemia and CIAF among children under five

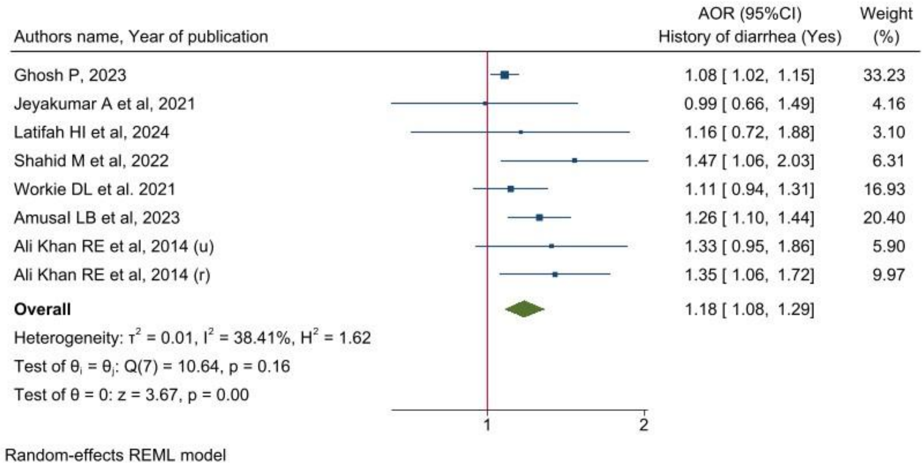

Fig C: Forest plot illustrating the relationship between a history of diarrhea in children and CIAF

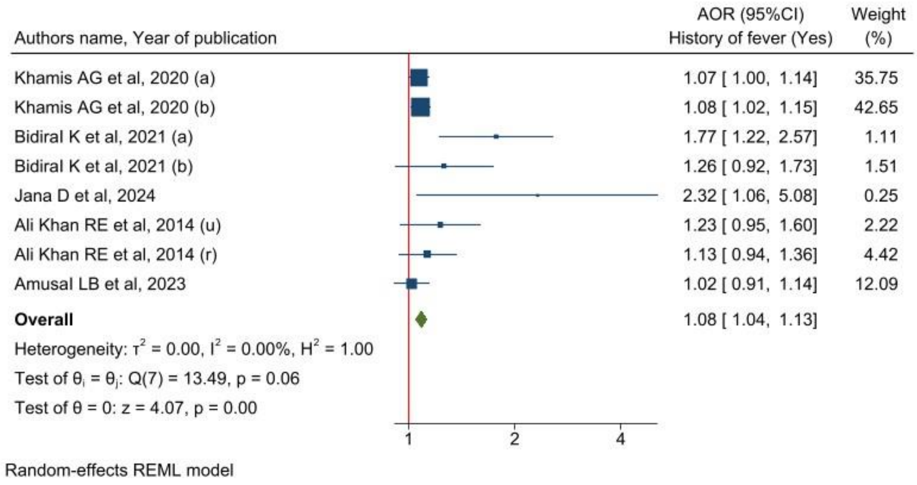

Fig D: Forest plot illustrating the relationship between a history of fever/ARI in children and CIAF

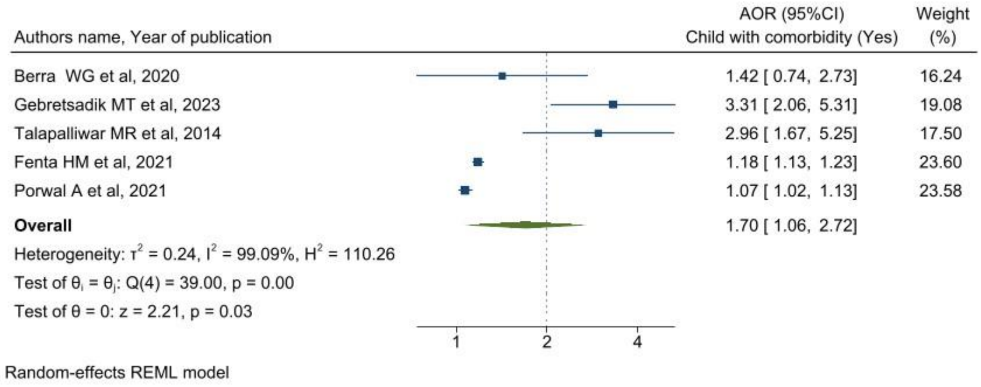

Fig E: Forest plot illustrating the relationship between comorbidity in children and CIAF

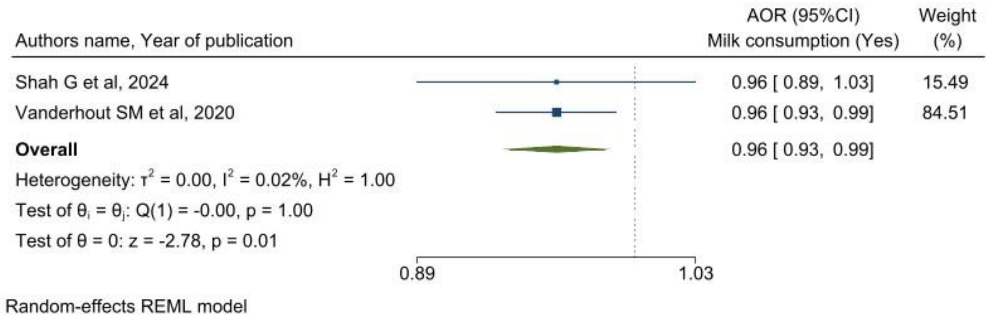

Fig F: Forest plot illustrating the relationship between milk consumption and CIAF

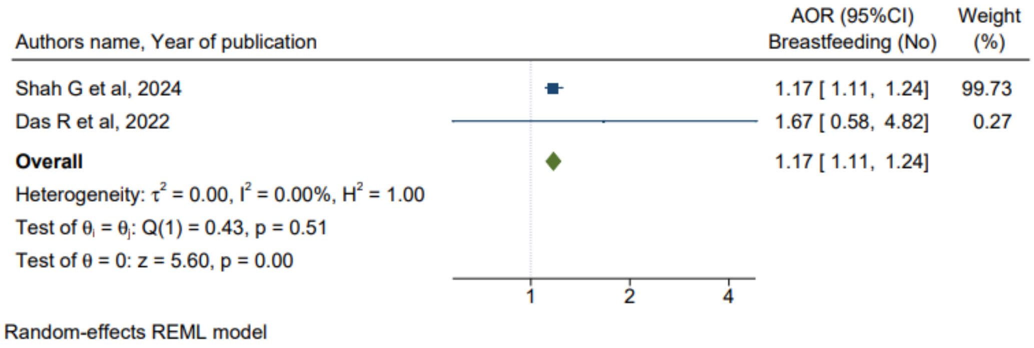

Fig G: Forest plot illustrating the relationship between breastfeeding and CIAF

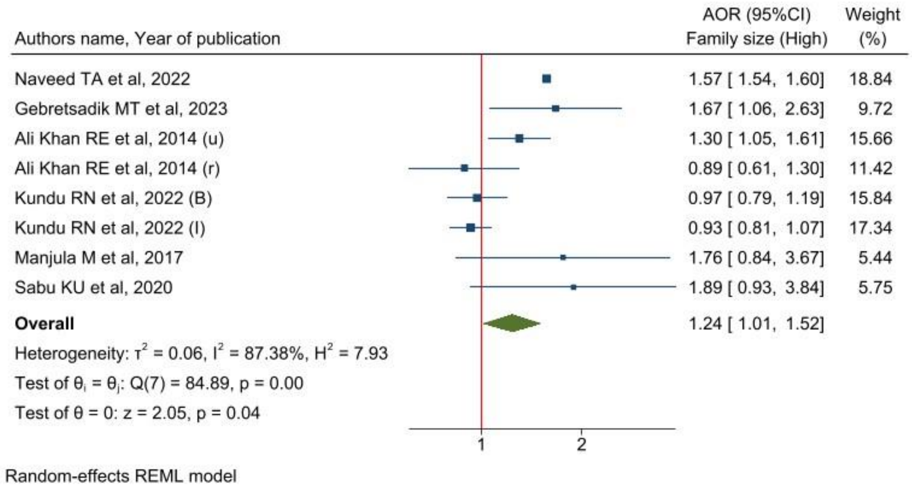

Fig H: Forest plot illustrating the relationship between family size and CIAF

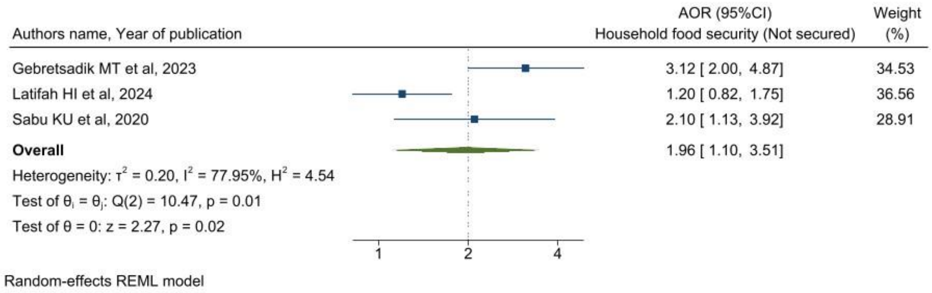

Fig I: Forest plot illustrating the association between household food security and CIAF among children under five

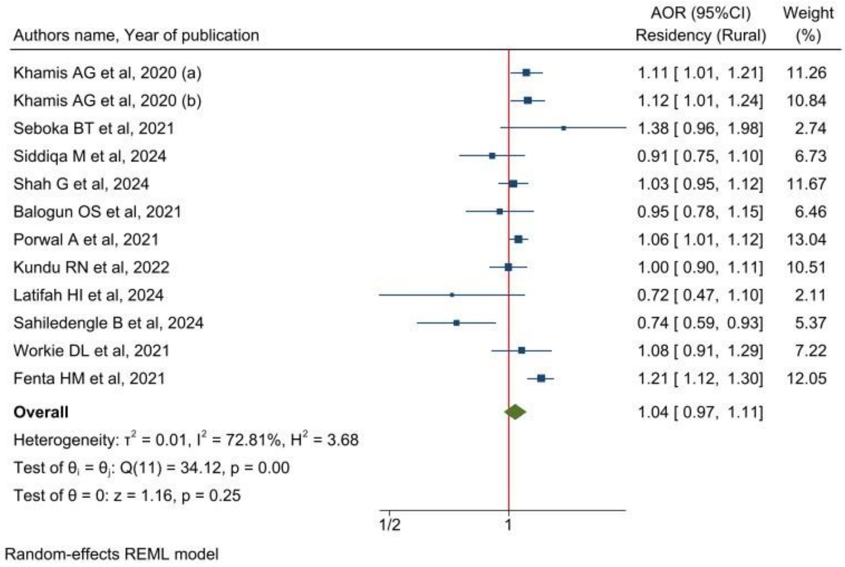

Fig J: Forest plot illustrating the association between place of residency and CIAF

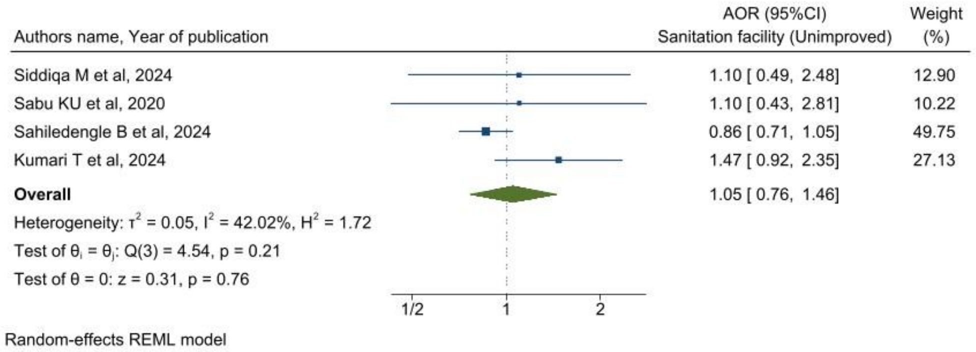

Fig K: Forest plot illustrating the association between sanitation facility and CIAF
