# Supplementary figures and images for: Socio-ecological determinants of multiple anthropometric failures among under-five children: A systematic review and meta-analysis of observational studies
Source: PLOS Glob Public Health. 2025 Jul 31;5(7):e0005008. doi: 10.1371/journal.pgph.0005008 (PMC12312983; doi:10.1371/journal.pgph.0005008)

**S2\_Fig: Funnel plots for assessing publication bias across studies**

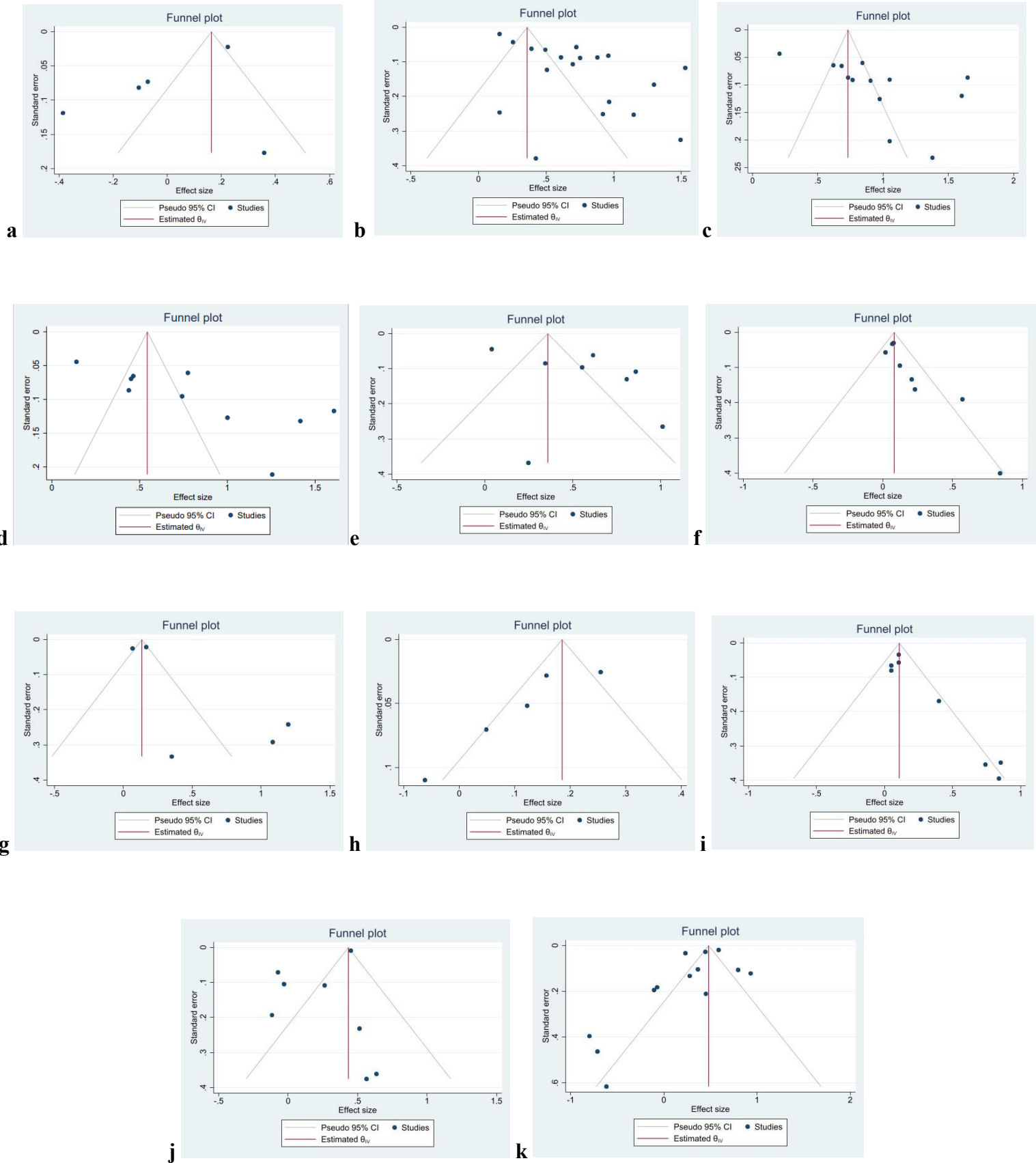

Supplement: S2 Fig — (PDF) [file pgph.0005008.s006.pdf]
