# Supplementary material for: Socio-ecological determinants of multiple anthropometric failures among under-five children: A systematic review and meta-analysis of observational studies
Source: PLOS Glob Public Health. 2025 Jul 31;5(7):e0005008. doi: 10.1371/journal.pgph.0005008 (PMC12312983; doi:10.1371/journal.pgph.0005008)
Supplement: S3 Fig — (PDF) [file pgph.0005008.s008.pdf]

S3_Fig: A leave-one-out sensitivity analysis results

Contents

Fig A: Leave-one-out sensitivity analysis results on the association between child’s age (12-23 months) and CIAF 2

Fig B: Leave-one-out sensitivity analysis results on the association between child’s age (24-35 months) and CIAF 2

Fig C: Leave-one-out sensitivity analysis results on the association between child’s age (36-48 months) and CIAF 3

Fig D: Leave-one-out sensitivity analysis results on the association between child’s age (49-59 months) and CIAF 3

Fig E: Leave-one-out sensitivity analysis results on the association between comorbidity and CIAF 3

Fig F: Leave-one-out sensitivity analysis results on the association between child's sex and CIAF 4

Fig G: Leave-one-out sensitivity analysis results on the association between history of fever and CIAF 4

Fig H: Leave-one-out sensitivity analysis results on the association between history of diarrhea and CIAF 4

Fig I: Leave-one-out sensitivity analysis results on the association between anemia and CIAF 5

Fig J: Leave-one-out sensitivity analysis results on the association between moderate/severe anemia and CIAF. 5

Fig K: Leave-one-out sensitivity analysis results on the association between birth weight and CIAF 5

Fig L: Leave-one-out sensitivity analysis results on the association between current breastfeeding and CIAF..... 6

Fig M: Leave-one-out sensitivity analysis results on the association between dietary diversity score and CIAF .. 6

Fig N: Leave-one-out sensitivity analysis results on the association between maternal education and CIAF 6

Fig O: Leave-one-out sensitivity analysis results on the association between maternal employment and CIAF ... 7

Fig P: Leave-one-out sensitivity analysis results on the association between family size and CIAF 7

Fig Q: Leave-one-out sensitivity analysis results on the association between wealth index and CIAF 7

Fig R: Leave-one-out sensitivity analysis results on the association between place of residence and CIAF 8

Fig S: Leave-one-out sensitivity analysis results on the association between drinking water sources and CIAF .. 8

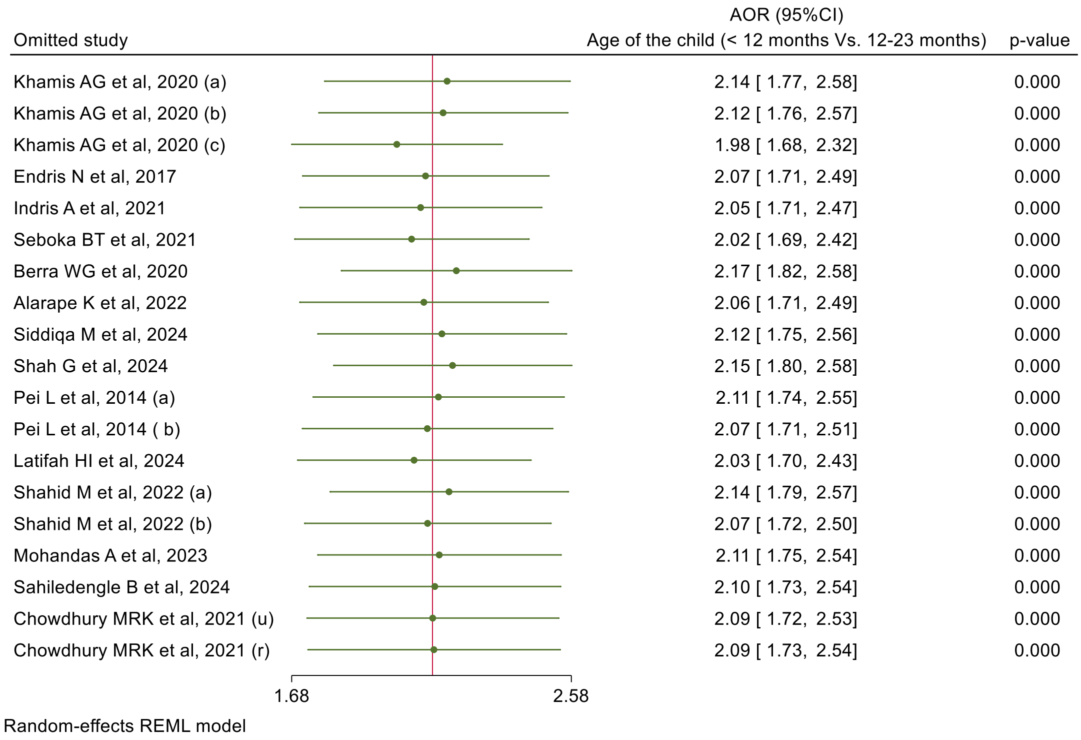

Fig A: Leave-one-out sensitivity analysis results on the association between child's age (12-23 months) and CIAF

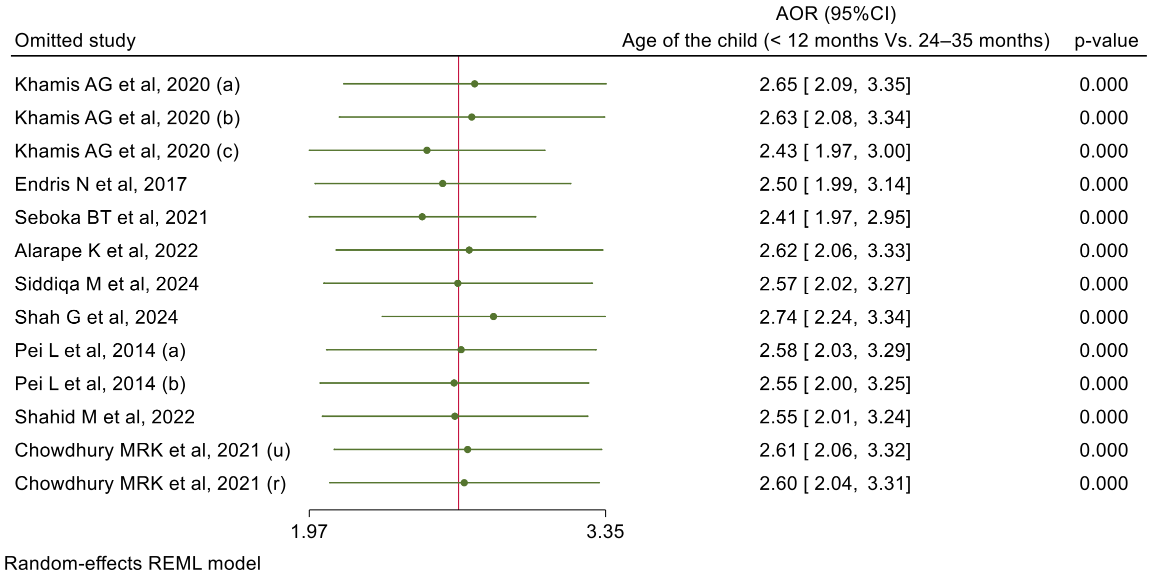

Fig B: Leave-one-out sensitivity analysis results on the association between child's age (24-35 months) and CIAF

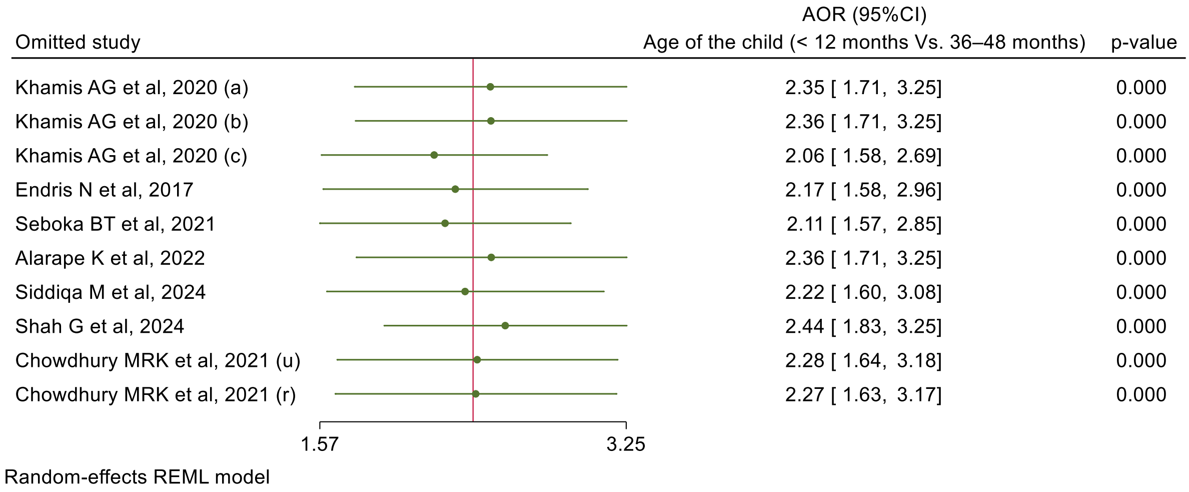

Fig C: Leave-one-out sensitivity analysis results on the association between child's age (36-48 months) and CIAF

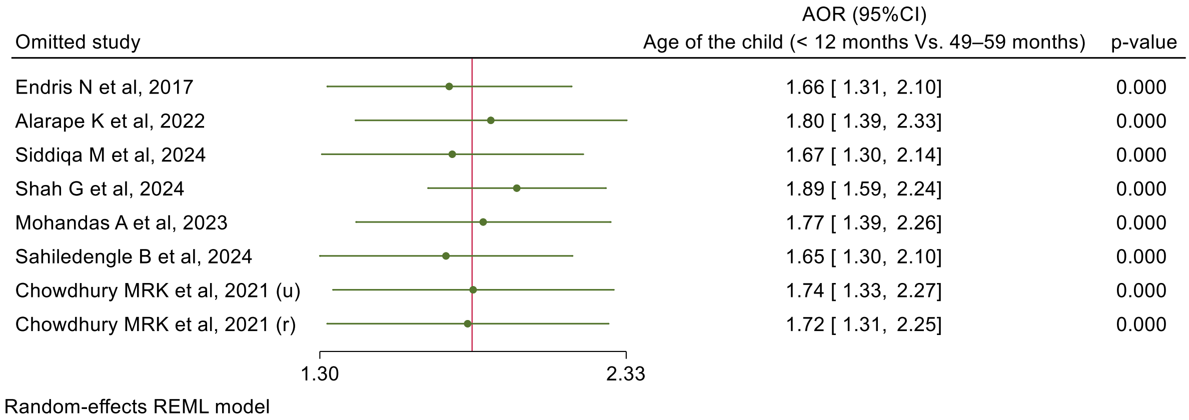

Fig D: Leave-one-out sensitivity analysis results on the association between child's age (49-59 months) and CIAF

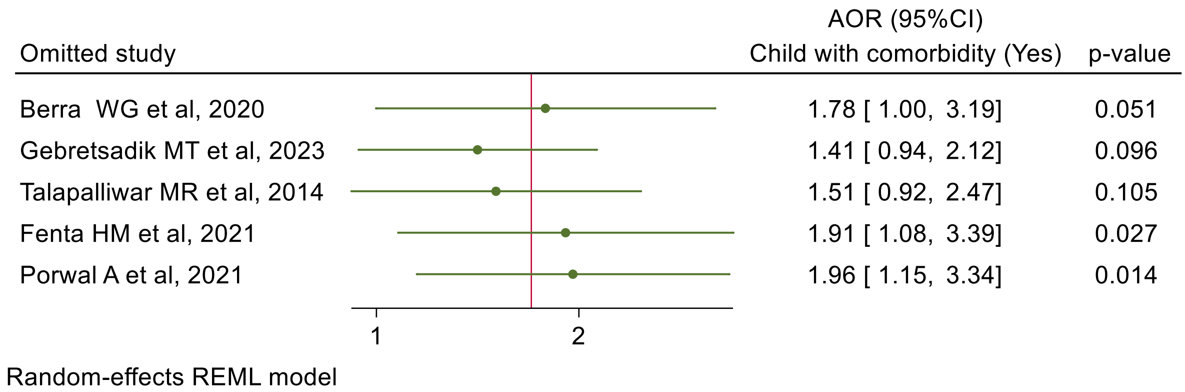

Fig E: Leave-one-out sensitivity analysis results on the association between comorbidity and CIAF

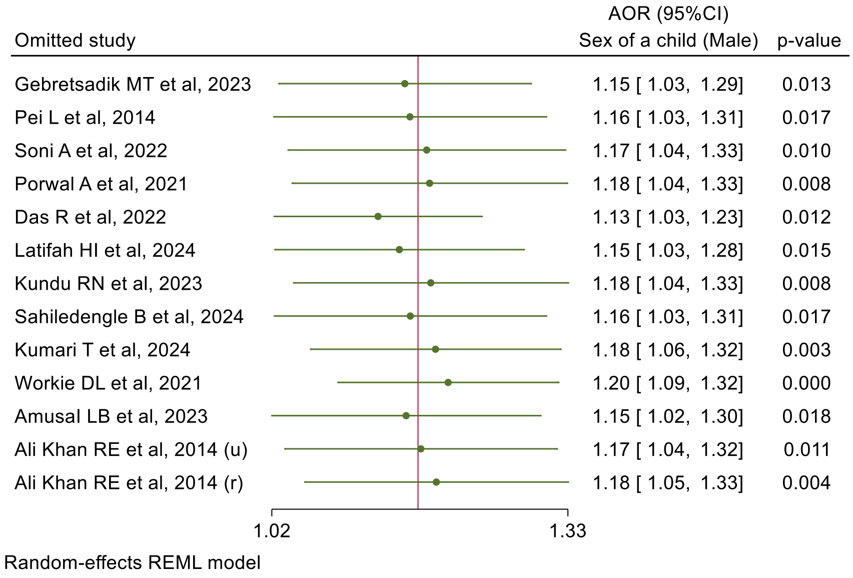

Fig F: Leave-one-out sensitivity analysis results on the association between child's sex and CIAF

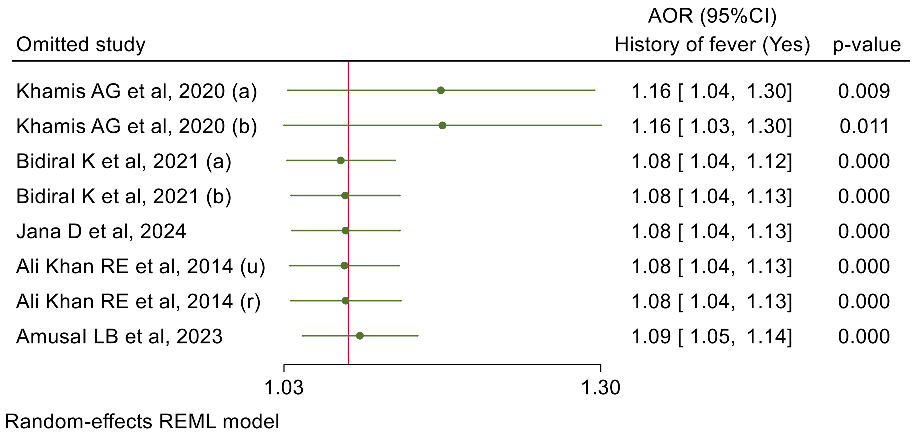

Fig G: Leave-one-out sensitivity analysis results on the association between history of fever and CIAF

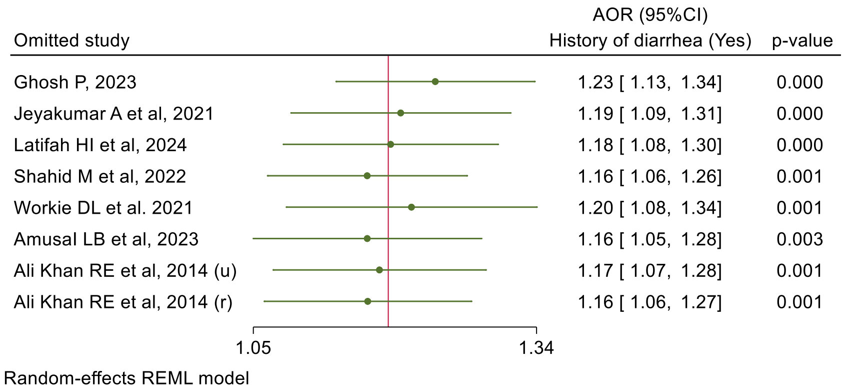

Fig H: Leave-one-out sensitivity analysis results on the association between history of diarrhea and CIAF

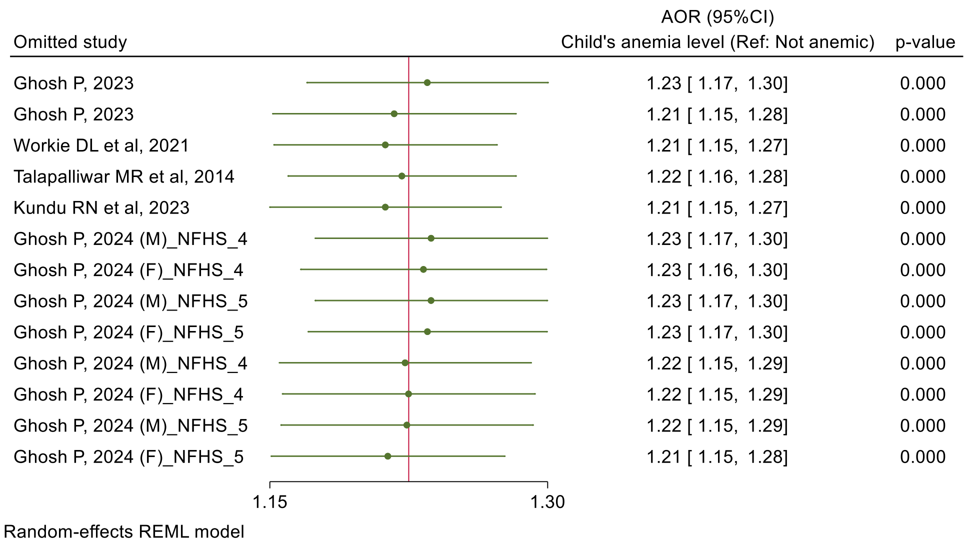

Fig I: Leave-one-out sensitivity analysis results on the association between anemia and CIAF

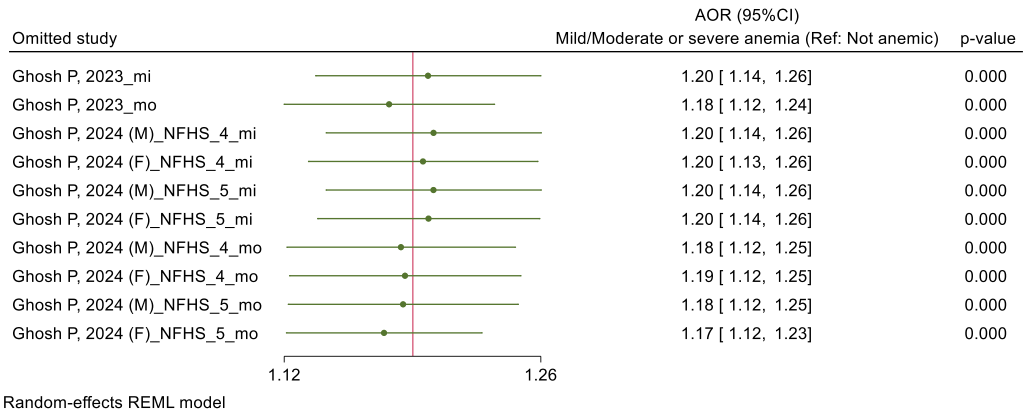

Fig J: Leave-one-out sensitivity analysis results on the association between moderate/severe anemia and CIAF

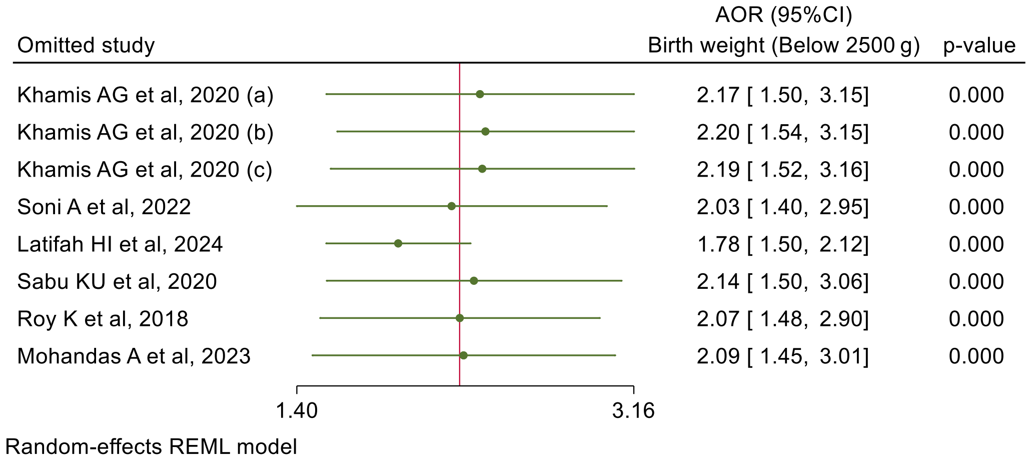

Fig K: Leave-one-out sensitivity analysis results on the association between birth weight and CIAF

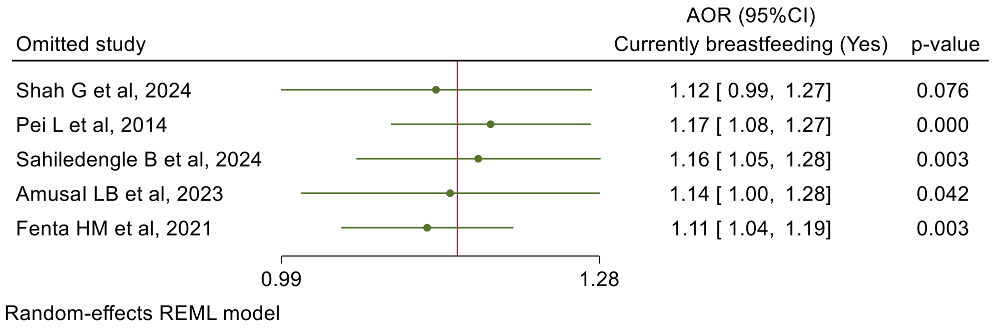

Fig L: Leave-one-out sensitivity analysis results on the association between current breastfeeding and CIAF

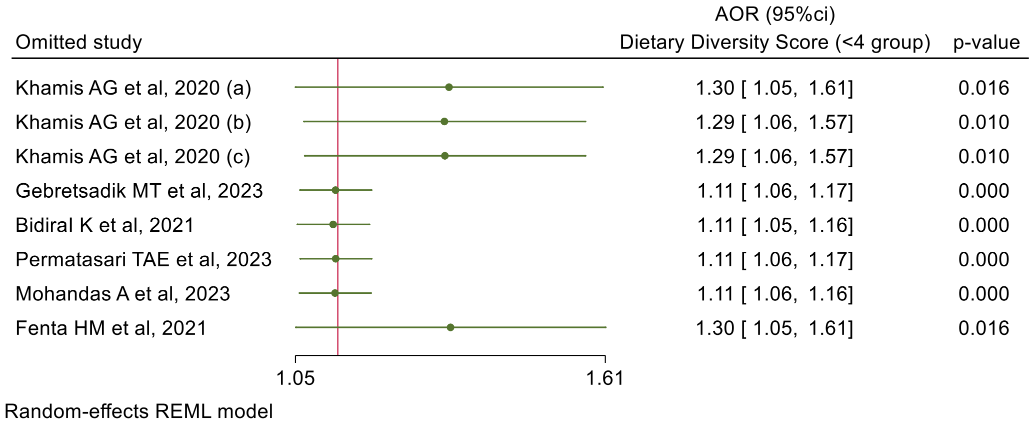

Fig M: Leave-one-out sensitivity analysis results on the association between dietary diversity score and CIAF

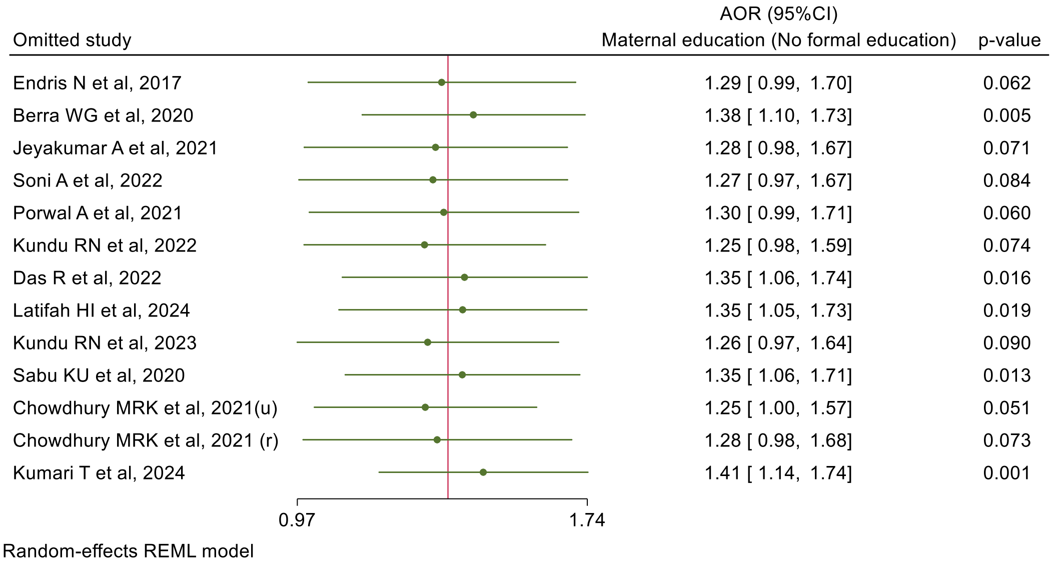

Fig N: Leave-one-out sensitivity analysis results on the association between maternal education and CIAF

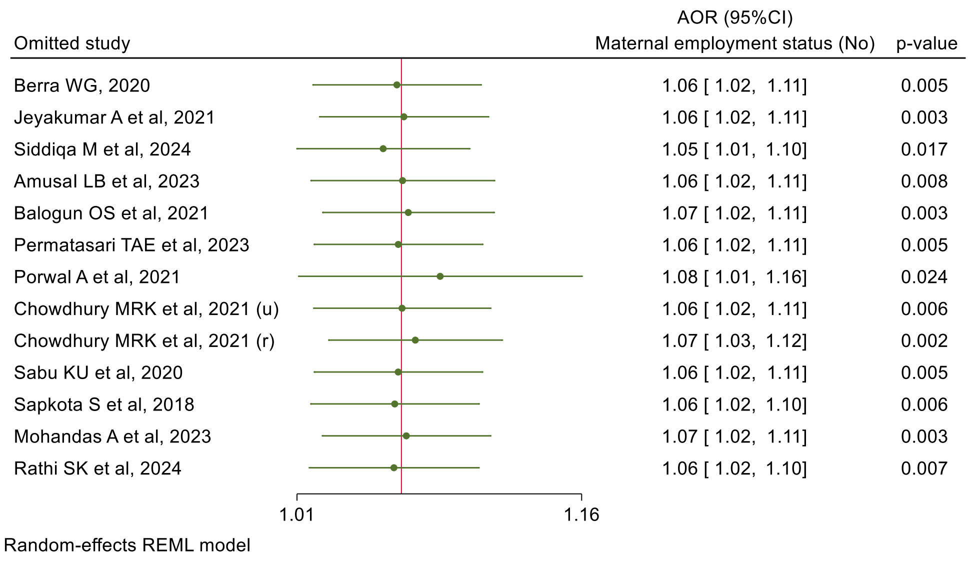

Fig O: Leave-one-out sensitivity analysis results on the association between maternal employment and CIAF

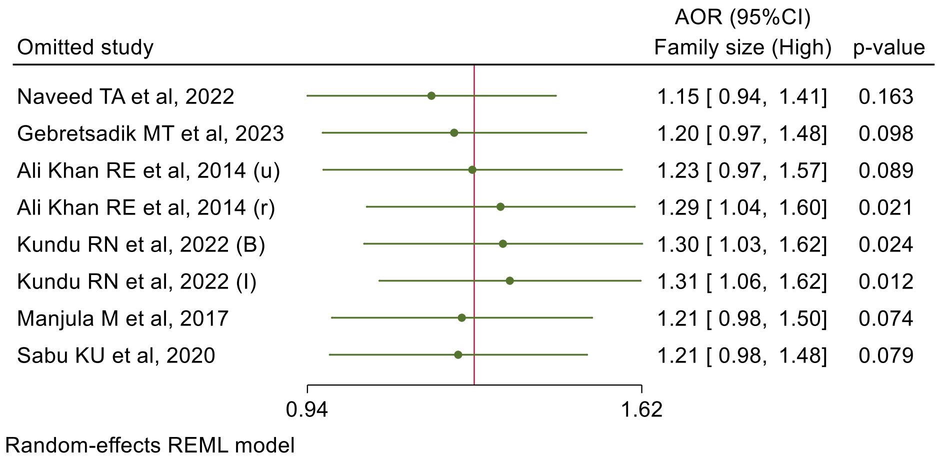

Fig P: Leave-one-out sensitivity analysis results on the association between family size and CIAF

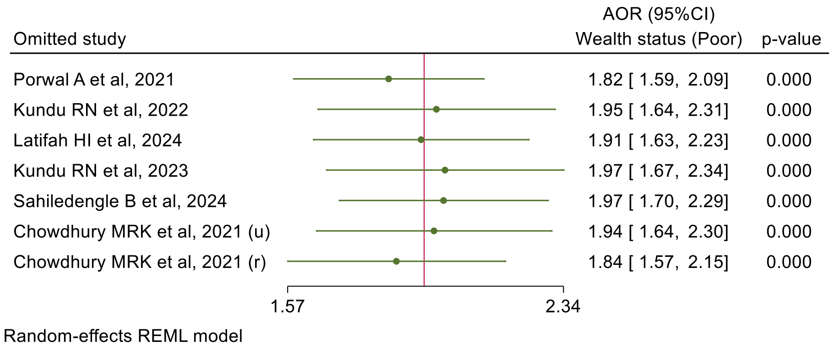

Fig Q: Leave-one-out sensitivity analysis results on the association between wealth index and CIAF

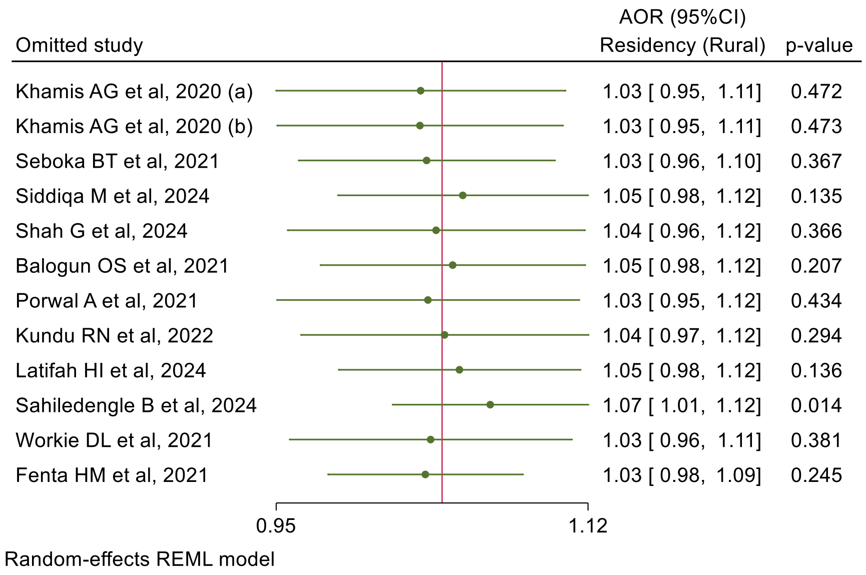

Fig R: Leave-one-out sensitivity analysis results on the association between place of residence and CIAF

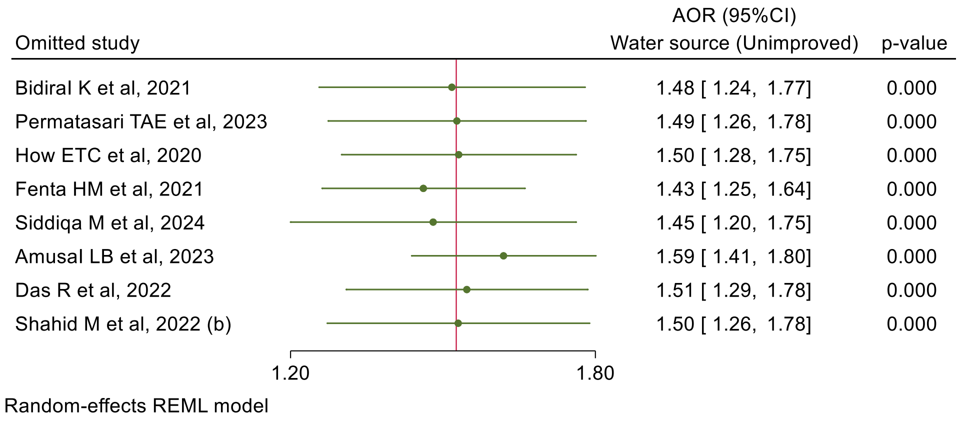

Fig S: Leave-one-out sensitivity analysis results on the association between drinking water sources and CIAF
